# Paleodistributions and Comparative Molecular Phylogeography of Leafcutter Ants (*Atta* spp.) Provide New Insight into the Origins of Amazonian Diversity

**DOI:** 10.1371/journal.pone.0002738

**Published:** 2008-07-23

**Authors:** Scott E. Solomon, Mauricio Bacci, Joaquim Martins, Giovanna Gonçalves Vinha, Ulrich G. Mueller

**Affiliations:** 1 Section of Integrative Biology, The University of Texas at Austin, Austin, Texas, United States of America; 2 Department of Entomology, Smithsonian Institution, Washington, D. C., United States of America; 3 Center for the Study of Social Insects, São Paulo State University, Rio Claro, São Paulo, Brazil; University of Kent, United Kingdom

## Abstract

The evolutionary basis for high species diversity in tropical regions of the world remains unresolved. Much research has focused on the biogeography of speciation in the Amazon Basin, which harbors the greatest diversity of terrestrial life. The leading hypotheses on allopatric diversification of Amazonian taxa are the Pleistocene refugia, marine incursion, and riverine barrier hypotheses. Recent advances in the fields of phylogeography and species-distribution modeling permit a modern re-evaluation of these hypotheses. Our approach combines comparative, molecular phylogeographic analyses using mitochondrial DNA sequence data with paleodistribution modeling of species ranges at the last glacial maximum (LGM) to test these hypotheses for three co-distributed species of leafcutter ants (*Atta* spp.). The cumulative results of all tests reject every prediction of the riverine barrier hypothesis, but are unable to reject several predictions of the Pleistocene refugia and marine incursion hypotheses. Coalescent dating analyses suggest that population structure formed recently (Pleistocene-Pliocene), but are unable to reject the possibility that Miocene events may be responsible for structuring populations in two of the three species examined. The available data therefore suggest that either marine incursions in the Miocene or climate changes during the Pleistocene—or both—have shaped the population structure of the three species examined. Our results also reconceptualize the traditional Pleistocene refugia hypothesis, and offer a novel framework for future research into the area.

## Introduction

Tropical regions around the world are well known for their rich diversity of life. Yet, the reasons why the tropics harbor more species than temperate and arctic regions remain unclear [1], [2], [3], [4]. The Amazon Basin has been of particular interest in this matter, as it harbors perhaps the world's greatest terrestrial biodiversity [5], [6], [7], [8]. As is true for the study of speciation in general [9], much focus has been placed on the biogeography of processes generating diversity in the Amazon Basin, specifically on how allopatry can be achieved in a landscape without obvious geographic barriers (although the presence of now invisible barriers, such as ancient “arches” has been suggested [10], [11], [12], [13]). Although a plethora of hypotheses have been suggested, three stand out as the most widely discussed. These are the Pleistocene refugia hypothesis, the marine incursion hypothesis, and the riverine barrier hypothesis.

The Pleistocene refugia hypothesis has been responsible for generating the most interest in the field [12], [13], [14], but has also become the most heavily criticized [15]. First proposed by Haffer in 1969 [16], this hypothesis suggests that historical climate changes, specifically during periods of glacial maxima, restricted the distribution of wet forests in Amazonia. Under this model, species that inhabited these forests (birds in Haffer's original hypothesis but later expanded to include all terrestrial species [12]) would likewise have become more isolated, resulting in the possibility for allopatric speciation. Haffer [16] proposed the presence of several Pleistocene forest refugia along the periphery of the Amazon Basin, reasoning that these mountainous regions would have enough surface relief to remain moist, even during periods of widespread aridity, by generating local precipitation [17].

Although some studies [18], [19], [20], [21], [22] have found support for the predictions of the Pleistocene refugia hypothesis (see Table 1 for a list of predictions), most have not [23], [24], [25], [26], [27]. Furthermore, the refugia hypothesis has been criticized because (1) geological and paleoclimatic data do not generally support the conclusion that wet forests were highly fragmented during the Pleistocene [15], [28], [29], [30]; (2) the locations and size of forest refugia, if they did exist, would be different for each species because of different environmental tolerances [12], [14]; (3) some areas that have been proposed as refugia because they appear to contain greater species diversity can be explained as artifacts of sampling biases [31]; and (4) the ages of many extant Amazonian species pre-date the Pleistocene, suggesting they were generated by earlier mechanisms [12], [14], [32]. These criticisms have led some researchers to call for the complete dismissal of the Pleistocene refugia hypothesis on the grounds that it has been sufficiently discredited [15].

**Table 1:** Summary of the predictions of each hypothesis and overview of the methods used to test them (*Diversification any time subsequent to the formation of the Amazon river (5–12 mya) would be consistent with the riverine barrier hypothesis, therefore only diversification prior to 12 mya would falsify this prediction; we chose not to use this as a test of the riverine barrier hypothesis because it is nearly impossible to reject for these species, which originated no more than 14 mya [81].).

Predictions	Pleistocene refugia	Marine incursion	Riverine barrier	Methods used
Reciprocal monophyly of populations:	in different refugia	in Eastern base of Andes, Brazilian Shield, and/or Guiana Shield	on opposite banks of Amazon River	Parametric bootstrap, Bayesian hypothesis tests
Basal populations are located:	in refugia	in Eastern base of Andes, Brazilian Shield, and/or Guiana Shield	N/A	ML and Bayesian gene tree reconstruction
Derived populations are located:	outside refugia	in Amazonian lowlands	N/A	ML and Bayesian gene tree reconstruction
Barrier to gene flow:	areas between refugia	Amazonian lowlands	Amazon River	AMOVA, Mantel tests
Population history includes:	bottlenecks and expansion	bottlenecks and expansion	N/A	Mismatch distributions, Tajima's D
Population structure formed:	during Pleistocene (10 kya–1.8 mya)	during Miocene (10–15 mya)	N/A*	IM

The marine incursion hypothesis stems from evidence that tectonic events combined with elevated sea levels, most recently during the mid-Miocene (approximately 10–15 mya), flooded much of the Amazon Basin in salty or brackish water [33], [34], [35], [36], [37], [38]. Such incursions would have restricted all terrestrial organisms inhabiting the Amazon region to become isolated in areas of higher elevation, namely near the Andes to the west, the Guiana Shield to the north, and the Brazilian Shield to the south. Under this model, the resulting isolation would permit allopatric divergence of these populations. Support for the marine incursion hypothesis has so far been found in woodcreepers [23] and freshwater fish [39].

The riverine barrier hypothesis can be traced to early observations on vertebrate distributions by Alfred Russell Wallace [40]. This hypothesis suggests that tropical rivers serve as barriers to gene flow for terrestrial organisms. These rivers, which are wide and numerous in Amazonia, may promote divergence of populations restricted to either side [14], [41], [42], [43], [44], [45]. This hypothesis has received mixed support. On the one hand, major Amazonian rivers do seem to restrict dispersal of passerine birds [46], small primates [47], lizards [48], [49], frogs [50] and Riodinid butterflies [51]. However, extensive molecular and morphological work on small mammals and frogs along the Juruá River, a tributary of the Amazon, failed to detect a significant river barrier effect [10], [41], [43], [44].

Two recent developments have allowed new insights into the predictions made by these hypotheses (see Table 1). First, advances in molecular techniques have not only increased the amount of data available for analysis, they also permit a more quantitative evaluation of species and population histories, which are essential for testing competing hypotheses on tropical diversification [14]. Although molecular reconstructions of the biogeography of past speciation events seems promising, the dynamic nature of species' geographic ranges makes these inferences somewhat tenuous [52]. An alternative approach is to examine the current population structure of widespread species. Such phylogeographic analyses can provide insight into the processes responsible for generating allopatry by giving not only a snapshot of the current population structure, but also a window into the past through the reconstruction of gene trees and historical demography [14], [53], [54].

The second recent development combines reconstructions of paleoclimates with a flurry of novel techniques for modeling species distributions under current as well as past (or future) climate conditions. Such “paleodistribution” analyses provide a means of independently assessing the extent to which past climate has influenced species' geographic ranges [55], [56], [57], [58], thereby avoiding assumptions about the presence and location of putative forest refugia and thus bypassing several of the major criticisms of the Pleistocene refugia hypothesis.

Several recent studies have demonstrated the utility of combining molecular phylogeography and paleodistribution reconstruction in a complimentary fashion to test a priori biogeographic hypotheses [55], [59], [60], [61]. However, paleoclimate data for the Amazon basin are not nearly as complete as for other regions, such as the Australian Wet Tropics [62], so such an approach has not yet been utilized for Amazonian species. Furthermore, the few studies that have used a molecular phylogeographic approach to test these supposedly universal hypotheses have primarily focused on vertebrate taxa [10], [23], [27], [43], [44], [45], [63], which represent only a small proportion of the total diversity of the Amazonian region [5], [6], [7], [64].

We used three co-distributed species of leafcutter ants in the genus *Atta* (Formicidae: Attini) to test the Pleistocene refugia, marine incursion, and riverine barrier hypotheses using a combination of paleodistribution modeling and comparative molecular phylogeography. Leafcutter ants are widespread throughout the Neotropics [65], [66]. They are generalist herbivores, cutting fresh vegetation as a food source for their mutualistic fungal gardens [67], [68]. Due to their tendency to forage on crops and ornamental plants [69], leafcutter ants are considered to be major agricultural pests, and have been described as the dominant herbivores of the Neotropics [66], [70]. They also play a key ecological role in nutrient cycling as they bring organic material deep into their subterranean nests [71], [72].

Three leafcutter ant species, *A. cephalotes*, *A. sexdens*, and *A. laevigata*, are ideal for testing the hypotheses in question because (1) they co-occur throughout much of the Amazon Basin, as well as in adjacent areas [65], [73], (2) they diversified within the relevant time frame for the hypotheses in question [74], (3) the three species differ in their environmental tolerances [75], [76], permitting an evaluation of how historical climatic changes have differentially influenced each, and (4) they can be easily collected due to their enormous colony sizes [66], [77].

We used these three species as independent tests of the predictions of each hypothesis (summarized in Table 1). Furthermore, we hypothesized that, since these species have similar distributions, dispersal abilities, and life histories [65], [73], [75], [76], [78], [79], [80], the riverine barrier hypothesis and marine incursion hypothesis should both apply equally to all three species. However, because the three species chosen in this study display a continuum of tolerance to aridity, such that *A. cephalotes* is the least tolerant of aridity, *A. laevigata* the most tolerant, and *A. sexdens* intermediate between the two [76], we hypothesized that each species would respond differently to historical climate change during the Pleistocene. Specifically, we predicted that increasing aridity during the Pleistocene, reaching a climax at the last glacial maximum (LGM; approximately 21,000 ybp) would have most restricted the distribution of the least arid-tolerant species, *Atta cephalotes*, while expanding the range of the most arid-tolerant, *Atta laevigata*, with *A. sexdens* affected to an intermediate extent. To test these predictions, we used a rigorous statistical framework combining paleodistribution modeling with gene tree reconstructions, population genetic analyses, historical demographic analyses, and coalescent dating analyses.

## Results

**Figure 1:**
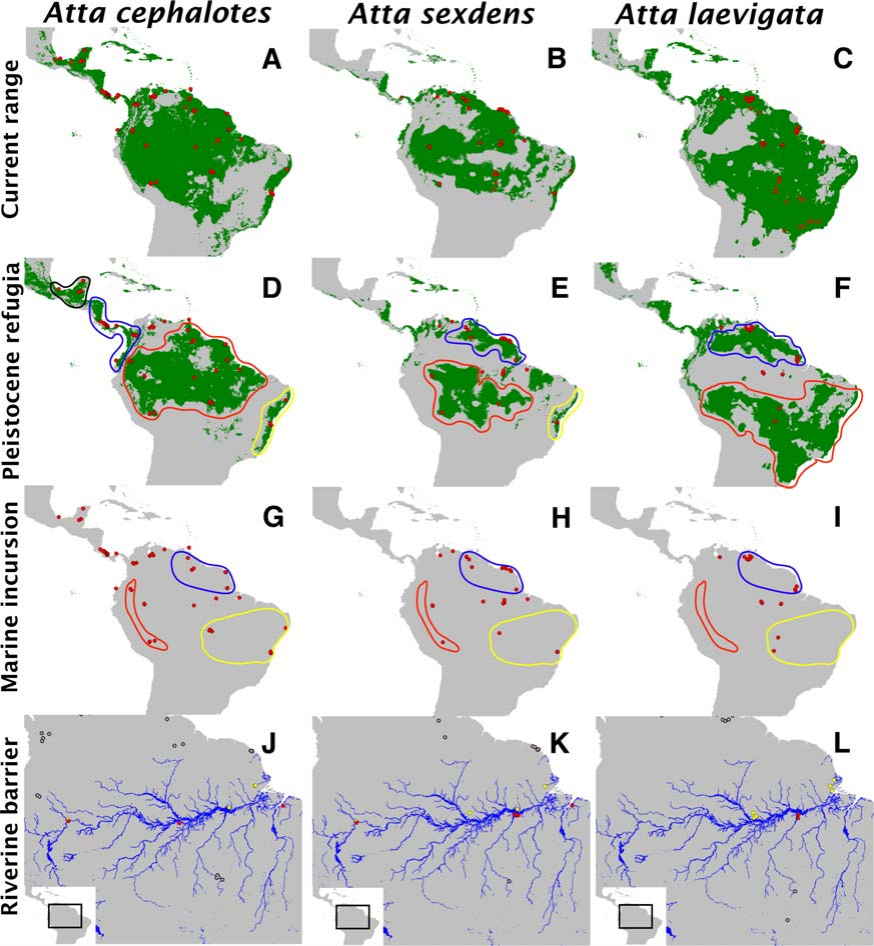
Overview of populations sampled and groupings used in hypothesis tests (left to right: *Atta cephalotes*, *Atta sexdens*, *Atta laevigata*). *A–C*: Results of maxent binary distribution models for the three species under current conditions. Areas predicted to be suitable for each species under current climate conditions are shaded in green. Populations used in this study are shown with red circles; populations for which molecular data were obtained are indicated by filled circles, while populations used only for distribution modeling are indicated by open circles. *D–F*: Paleodistributions of the three species at the LGM (21 kya) estimated by projecting the maxent model for current conditions onto climate layers from the LGM. Red circles indicate populations used in molecular analyses; Regions outlined with colored lines show population groupings used to test the Pleistocene refugia hypothesis. *G–I*: Population groupings used to test the marine incursion hypothesis are circled with colored lines (red = Andes, blue = Guiana Shield, yellow = Brazilian Shield); populations for which molecular data were obtained are indicated by filled circles. *J–L*: Populations used to test the riverine barrier hypothesis are shown with yellow or red circles, indicating populations located north or south of the Amazon river, respectively. Populations for which molecular data were obtained but are located away from the Amazon river (and are therefore not considered in tests of this hypothesis) are shown with empty, black circles.

Maps comparing the potential geographic range of each species under current conditions and during the last glacial maximum (LGM), approximately 21 kya, are shown in Figure 1. For current conditions, the area under the receiver operating characteristic curve (AUC) was 0.996, 0.983, and 0.986 for *A. cephalotes*, *A. laevigata*, and *A. sexdens*, respectively. Furthermore, out of the ten different thresholds (see Methods) used to obtain a binary (i.e. presence/absence) prediction, all ten were significantly better than random models for all three species. The cumulative probability thresholds (chosen such that they minimized the commission (false positive) rate for current conditions; see Methods) for *A. cephalotes*, *A. sexdens*, and *A. laevigata* were 1, 5, and 5, respectively.

The projected distribution of each species at the LGM is shown in panels D–F of Figure 1. Putative refugial areas, used for subsequent hypothesis testing, were defined as contiguous areas (i.e. solid green in Figure 1D–F) projected to have been suitable habitat for a given species at the LGM (areas within colored circles in Figure 1D–F). Areas predicted to have been suitable at the LGM, but for which no samples were obtained, were logically excluded for the purposes of hypothesis testing. For *A. cephalotes*, the potential LGM range spanned most of the Amazon Basin, with a contiguous population throughout the Guiana Shield (Figure 1D). This range is somewhat reduced from the estimated current potential distribution of the species (Figure 1A.). Other areas with high probability of occurrence during the LGM include the Atlantic Coastal Forests of Brazil, lower Central America and the Chocó region of South America west of the Andes, and upper Central America into central Mexico (the latter two regions are separated by an area, corresponding to modern day Honduras, predicted to have only very small patches of suitable habitat and was therefore not considered a refugium for hypothesis testing). For *A. sexdens*, the paleodistribution model predicts a more fragmented potential distribution during the LGM (Figure 1E). The largest block of inhabitable range was in the southwestern Amazon Basin, from approximately just west of Manaus to the southwestern edge of the Peruvian Andes. Other blocks of inhabitable areas during the LGM for *A. sexdens* include the Guiana Shield, the Atlantic Coastal Forests of Brazil, an area south of the mouth of the Amazon River roughly between Belem and São Luis, northwestern Colombia/eastern Panama, and Nicaragua. For *A. laevigata*, the model predicted the presence of a large area of unsuitable habitat spanning much of the Amazon Basin (Figure 1F). The remaining areas of suitable habitat occur to the north and south of the Amazon Basin, and are themselves somewhat fragmented.

**Figure 2:**
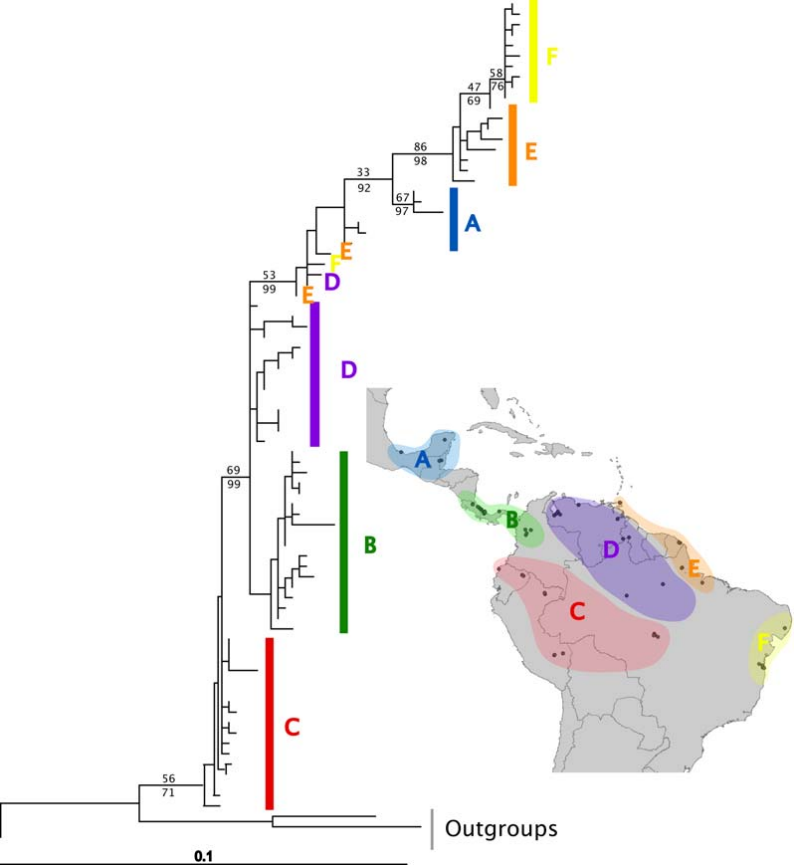
Maximum likelihood gene tree for *Atta cephalotes*. Support values are 100 ML Bootstrap (top) and Bayesian posterior probabilities (bottom). Outgroup sequences used for rooting were from *A. columbica*, *A. texana*, and *A. mexicana*. Uppercase letters correspond to regions shown on map.

The topologies of mitochondrial gene trees are shown in Figures 2–4. With one exception, these topologies were not consistent with reciprocal monophyly of the populations predicted by any of the three hypotheses, as determined by parametric bootstrap and Bayesian hypothesis tests (Table S2). The exception was the gene tree for *A. sexdens*, in which the populations predicted by the Pleistocene refugia hypothesis were reciprocally monophyletic (parametric bootstrap *p* = 0.15; Bayesian posterior probability = 0.843). However, the gene trees for *A. cephalotes* and *A. laevigata* did have the relevant basal and derived populations as predicted by both the Pleistocene refugia and marine incursion hypotheses. The gene tree for *A. sexdens* is split at the base into two reciprocally monophyletic clades that correspond to geographically distinct populations, such that no statement could be made about which populations are basal versus derived.

**Figure 3 pone-0002738-g003:**
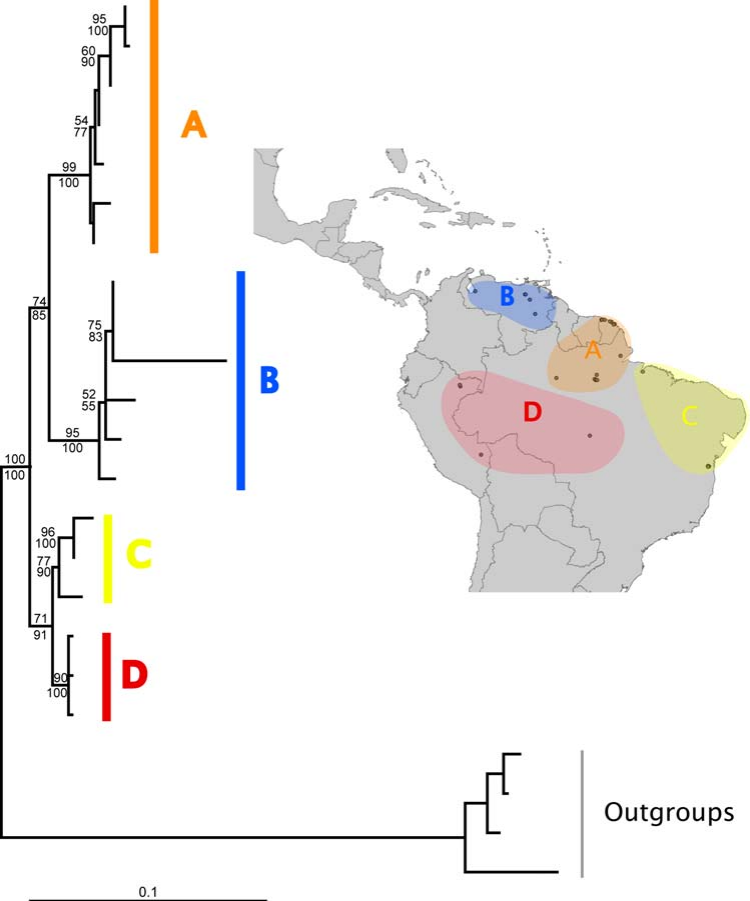
Maximum likelihood gene tree for *Atta sexdens*. Support values are 100 ML Bootstrap (top) and Bayesian posterior probabilities (bottom). Outgroup sequences used for rooting were from *A. laevigata*. Uppercase letters correspond to regions shown on map.

**Figure 4:**
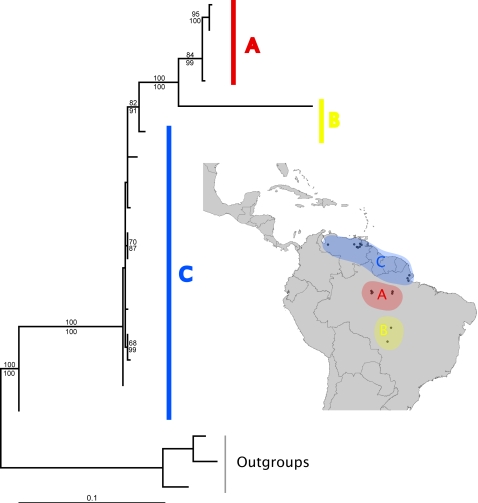
Maximum likelihood gene tree for *Atta laevigata*. Support values are 100 ML bootstrap replicates (top) and Bayesian posterior probabilities (bottom). Outgroup sequences used for rooting were from *A. sexdens*. Uppercase letters correspond to regions shown on map.

Population genetic analyses (AMOVA and Mantel tests) failed to find any evidence that the lower Amazon River has served as a barrier to gene flow for any of the three species (Tables S3–S4). For the Pleistocene refugia and marine incursion hypotheses, analyses of molecular variance (AMOVA) rejected the predicted barrier in all cases (Table S3) except for the barrier predicted by the Pleistocene refugia hypothesis for *A. cephalotes* (40.19% of variance explained by the refugia dictated by paleoclimate reconstructions; *p* = 0.00098). In contrast, partial Mantel tests (Table S4) could not reject the barrier predicted by the Pleistocene refugia or marine incursion hypotheses for any of the three species (*A. cephalotes*: marine incursion *r* = −0.149, *p* = 0.00003; Pleistocene refugia *r* = 0.076, *p* = 0.00589; *A. sexdens*: marine incursion *r* = −0.396, *p* = 0.00138; Pleistocene refugia *r* = 0.251, *p* = 0.00009; *A. laevigata*: marine incursion/Pleistocene refugia *r* = 0.472043, *p* = 0.0073).

Evidence for population bottlenecks and subsequent expansions was mixed in the two tests used (Table S5). For the purposes of discussion, an inference of population expansion was only made in the three instances in which both goodness-of-fit measures used to evaluate mismatch distributions, as well as Tajima's *D* statistic, were all consistent with population expansion (*A. cephalotes*, Pleistocene refugia: Atlantic Coast population [SSD = 0.0200829, *p* = 0.299; Harpending's Raggedness Index = 0.08930211, *p* = 0.3; Tajima's *D* = −1.65893, *p* = 0.033]; *A. sexdens*, marine incursion: Brazilian Shield population [SSD = 0.20368588, *p* = 0.137; Harpending's Raggedness Index = 0.47, *p* = 0.191; Tajima's *D* = −1.21852, *p* = 0.026]; *A. laevigata*, marine incursion/Pleistocene refugia: Guiana Shield population [SSD = 0.01959799, p = 0.181; Harpending's Raggedness Index = 0.10577614, p = 0.212; Tajima's *D* = −2.31554, *p* = 0]). In all other instances, at least one statistic was inconsistent with population expansion, or there were insufficient data.

**Figure 5:**
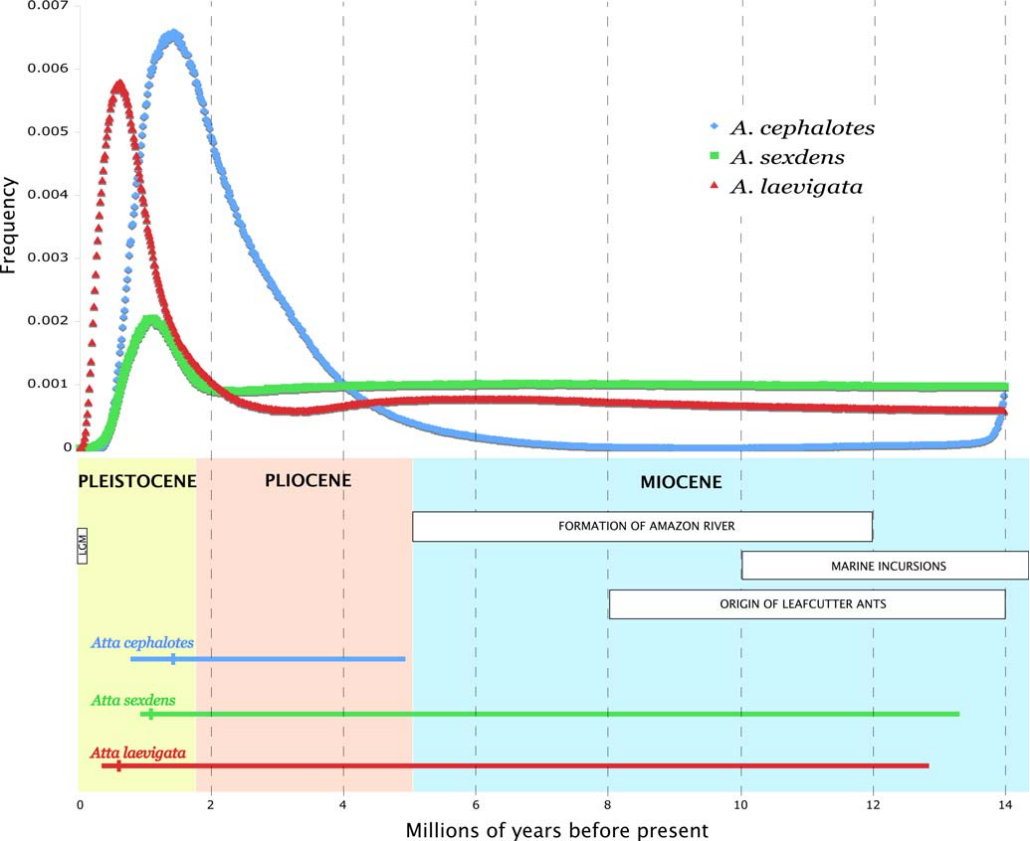
Timeline of diversification in Amazonian *Atta* species. *Top*: Posterior distributions of T_div_, the time since the oldest population division for each species as reconstructed for each species using the program IM. *Bottom*: The 95% confidence limits for diversification in each species are represented by horizontal bars, with a vertical bar indicating the best estimate for T_div_, assuming a mutation rate of 9.5 substitutions per site per million years and a generation time of 4 years.

Coalescent dating analyses that estimated the oldest measurable split (T_div_) between extant populations for each species are shown in Figure 5. The mode, upper, and lower 95% confidence intervals of T_div_ are given in Table S6. In all three species, the posterior distribution of T_div_ has a peak within the Pleistocene, and a long tail that extends into the Pliocene and/or Miocene. The long tail results in a rather wide 95% confidence interval, and is partially due to the high value (t_max_ = 133, corresponding to 14 mya) used as an upper bound for the time since divergence in all three species. This value was chosen based on the results of dating analyses for the tribe Attini, in which the crown group of leafcutter ants were estimated to have originated between 8 and 14 mya [81]. The value for t_max_ used in this study is thus somewhat conservative and likely extended the 95% confidence interval farther than would a lower value; however, given the data currently available, it would not be justified to use a lower value for t_max_.

The 95% confidence interval for population divergence in *Atta cephalotes* extends from the mid-Pleistocene (819 kya) to the lower Pliocene (4.893 mya), but does not include the Miocene (Figure 5). It therefore appears that the population structure currently present in *A. cephalotes* formed too recently to be explained by marine incursions during the Miocene. For the other two species, however, the upper 95% confidence limit (13.279 mya and 12.817 mya in *A. sexdens* and *A. laevigata*, respectively) extends into the Miocene, including the period between 10 and 15 mya when marine incursions into the Amazon Basin are thought to have achieved their greatest extent [38]. The wider confidence interval in these two species may also be due to the smaller sample sizes for *A. sexdens* (N = 46) and *A. laevigata* (N = 30) compared with *A. cephalotes* (N = 118).

## Discussion

Combining the results of the paleodistribution models with the molecular phylogeographic analyses (Table 2), the accumulated data rejected every prediction of the riverine barrier model for all three species examined. The results of AMOVA and Mantel tests for the presence of barriers to gene flow, as well as the topologies of mitochondrial gene trees (in which closely related haplotypes are found on opposite river banks), suggest that gene flow regularly occurs across the lower Amazon River in all three species. Although the exact dispersal abilities of *Atta* species are not known, typical flight distances for mated queens are thought to be less than 2 km (Mueller, pers. obs.), with a maximum range of no more than 50 km [82]. The main channel of the lower Amazon river (e.g. near the city of Santerém) is between 1 and 3 km in width, although the seasonal floodplain can be 20 to 40 km wide in the wet season [83]. The floodplain width is probably more relevant as a dispersal barrier to leafcutter ants since they do not survive in seasonally inundated soils (Solomon, pers. obs.). Although the potential barrier effects of other major rivers in the Amazon Basin were not tested in this analysis, the lack of a significant effect of the lower Amazon River suggests that smaller rivers are unlikely to structure populations of leafcutter ants.

**Table 2:** Overview of results.

Species	Prediction	Test	Riverine barrier	Pleistocene refugia	Marine incursion
*A. cephalotes*	Reciprocal monophyly of relevant populations	Parametric bootstrap	1/1	1/1	1/1
		Bayesian hypothesis tests	1/1	1/1	1/1
	Relevant basal and derived populations	ML and Bayesian trees	N/A	0/1	1/1
	Evidence for predicted barrier to gene flow	AMOVA	1/1	0/1	1/1
		Mantel Tests	1/1	0/1	0/1
	History of population expansions	Mismatch Distributions	N/A	1/8	0/6
		Tajima's *D*	N/A	2/4	3/3
	Appropriate age of oldest population division	IM	N/A	0/1	1/1
*A. sexdens*	Reciprocal monophyly of relevant populations	Parametric bootstrap	1/1	0/1	1/1
		Bayesian hypothesis tests	1/1	0/1	1/1
	Relevant basal and derived populations	ML and Bayesian trees	N/A	*	*
	Evidence for predicted barrier to gene flow	AMOVA	1/1	1/1	1/1
		Mantel Tests	1/1	0/1	0/1
	History of population expansions	Mismatch Distributions	N/A	3/6	1/4
		Tajima's *D*	N/A	3/3	1/2
	Appropriate date for oldest population division	IM	N/A	0/1	0/1
A. laevigata**	Reciprocal monophyly of relevant populations	Parametric bootstrap	1/1	1/1 **	1/1 **
		Bayesian hypothesis tests	1/1	1/1 **	1/1 **
	Relevant basal and derived populations	ML and Bayesian trees	N/A	0/1 **	0/1 **
	Evidence for predicted barrier to gene flow	AMOVA	1/1	1/1 **	1/1 **
		Mantel Tests	1/1	0/1 **	0/1 **
	History of population expansions	Mismatch Distributions	N/A	0/2 **	0/2 **
		Tajima's *D*	N/A	1/2 **	1/2 **
	Appropriate date for oldest population division	IM	N/A	0/1 **	0/1 **

The number of instances (statistical tests per species or per population) in which the relevant prediction could be rejected are indicated followed after a slash by the total number of instances (e.g. 1/2 means that one out of the two tests rejected the prediction); predictions which are not applicable are indicated by “N/A” (^*^the gene tree for *A. sexdens* could not resolve which populations were basal or derived; ^**^the predictions for the Pleistocene refugia and marine incursion models are identical for *A. laevigata*).

In contrast, discriminating between the Pleistocene refugia and marine incursion hypotheses was more difficult. This difficulty is due in part to the similar predictions that these hypotheses make (Table 1), since some areas reconstructed as refugia are also areas that would have avoided flooding during marine incursions (Figure 1) [39], [84], [85]. However, by reconstructing the paleodistribution of each species independently, our approach to testing the Pleistocene refugia hypothesis avoids this issue (in part; see below) since (1) we do not make the assumption that only areas of high surface relief served as refugia, and (2) the areas reconstructed as refugia are different for each species whereas the areas that avoided marine incursions are the same for each species. Nevertheless, the reconstructed paleodistribution for *Atta laevigata* at the LGM (Figure 1F) coincides with the areas unaffected by marine incursions (i.e. the Brazilian Shield and the Guiana Shield), so the predictions of these two hypotheses were largely identical for this species.

Paleodistribution modeling of species ranges during the LGM also addresses one of the major criticisms of the Pleistocene refugia hypothesis, namely that the locations and size of putative forest refugia are likely to be different for every species considered [12], [14]. The results of paleodistribution models in the current study strengthen this argument, since each of the congeneric species examined is predicted to have responded differently to environmental conditions at the LGM (Figure 1). Interestingly, the paleoclimate model used in this study predicts that conditions supporting wet forests persisted throughout much of the Amazon Basin during the LGM, as is suggested by an increasing amount of fossil pollen and other geological information [15], [29]. However, this reconstruction of Pleistocene climate conditions contradicts claims by proponents of the refugia model that wet forest only existed along the margins of the Amazon Basin during the LGM [13], [16], [17], [86].

The molecular data provided mixed support for the predictions of the Pleistocene refugia and the marine incursion hypotheses (Table 2). Reciprocal monophyly of the relevant populations was only found in one instance: the gene tree of *Atta sexdens* as predicted by the Pleistocene refugia hypothesis. However, failure to detect reciprocal monophyly does not necessarily indicate that the predictions of a given hypothesis have been invalidated, since incomplete lineage sorting is expected to also produce paraphyletic and polyphyletic gene trees as populations are split by barriers to gene flow [87]. Thus, although detecting reciprocal monophyly provides strong support for the predicted genealogical history of a species, failure to detect it does not necessarily indicate that the relevant populations are not diverging in the expected manner, especially if the suspected barrier promoting divergence appeared recently. Visual inspection of the gene tree topologies (Figures 2–4) offers an alternative way of interpreting a species genealogical history that is less sensitive to the effects of incomplete lineage sorting. Such an approach shows that the populations expected to be more basal and/or more derived did in fact occupy the positions predicted for *Atta cephalotes* by the Pleistocene refugia hypothesis, but not the marine incursion hypothesis. The gene tree for *Atta laevigata* shows the predicted positions for both these hypotheses (which make identical predictions, as explained above). However, because of the reciprocal monophyly of the relevant populations of *Atta sexdens*, the gene tree could not determine which populations were more basal or derived for this species.

The Pleistocene refugia and marine incursion hypotheses predict that areas that were historically unsuitable for a species to occur (due to inappropriate climatic conditions for the former, flooded areas for the latter) formed barriers to gene flow. The two methods used to test for the presence of these barriers (AMOVA and Mantel tests) did not always provide congruent results (Table 2, and Tables S3−S4). In fact, the Mantel tests failed to reject the barrier of interest in all six instances, while the AMOVA found no evidence for the barrier of interest in the case of the marine incursion hypothesis for *Atta cephalotes*, both the marine incursion and Pleistocene refugia hypotheses for *Atta sexdens*, and both hypotheses (which, again, make identical predictions) for *Atta laevigata*. It would therefore appear that the AMOVA is a more sensitive way of testing for the presence of gene flow barriers, although we are not aware of any studies that directly compare the discriminatory power of these two commonly used tests.

The two methods used to test for population bottlenecks and subsequent expansions, as predicted by both the Pleistocene refugia and marine incursion hypotheses, also occasionally gave conflicting results (Table 2). We interpreted the results in a conservative manner, such that population bottlenecks and expansions were only inferred in instances in which the results were unanimously consistent with such a demographic history. This was the case in three instances: one population (“Atlantic Coast”) of *A. cephalotes* predicted by the Pleistocene refugia hypothesis, one population (“Brazilian Shield”) of *A. sexdens* predicted by the marine incursion hypothesis, and one population (“Guiana Shield”) of *A. laevigata* predicted by both hypotheses. The results of these tests therefore do not provide much assistance in discriminating between the Pleistocene refugia and marine incursion hypotheses.

Despite the largely similar predictions about geographic population structure made by the Pleistocene refugia and marine incursion hypotheses, these hypotheses operate on vastly different temporal scales. On the one hand, marine incursions are thought to have taken places during the Miocene [33], [34], [37], [39], [84], (approximately 10–15 mya), whereas conditions thought to promote refugia existed, generally speaking, during the Pleistocene (1.8 mya–10 kya) and reached a climax during the last glacial maximum (21 kya) [13], [16], [17], [18], [88]. The results of our coalescent dating analyses indicate that the population structure observed today in all three species formed between 371,000 and 13.279 million years ago, a time period spanning the Pleistocene, Pliocene, and late Miocene. The 95% confidence interval for population divergence in *A. cephalotes* does not include the Miocene, suggesting that marine incursions are unlikely to have been responsible for forming the population structure observed today in this species. However, despite a trend toward diversification during the Pleistocene in all three species (Figure 5), marine incursions could not be ruled out as the source of population divergence in *A. sexdens* or *A. laevigata*. More precise dating of the origin of each of these species will require a species-level phylogenetic analysis of the leafcutter ants as well as their closest living relatives, *Trachymyrmex*, which have fossils that can provide calibration points [81].

Although our results are not able to differentiate between many of the predictions of the Pleistocene refugia and marine incursion hypotheses, except possibly for *A. cephalotes*, it is important to recognize, as some other authors have also noted [12], [23], [25], [50], that these hypotheses are not necessarily mutually exclusive. Marine incursions in the Miocene could have been followed by isolation into refugia during the Pleistocene, with species present at both times being affected by both. It is therefore possible that our inability to differentiate between these two “competing” hypotheses may be more a function of their inherent compatibility than their mutual exclusivity.

It should also be noted that, despite some support for the Pleistocene refugia hypothesis, our results do not support its traditional formulation, namely that isolated pockets of wet forest at the periphery of the Amazon Basin were the refugia responsible for diversification in all species [13], [17]. Instead, the results of our paleodistribution reconstructions, and to a large extent the genetic data, suggest that the species restricted to wet forest, *A. cephalotes*, was the most widespread at the LGM, while the species most closely associated with open habitat, *A. laevigata*, was the most fragmented. This result is exactly opposite that predicted by the traditional Pleistocene refugia hypothesis, and suggests a more general model for the role of Pleistocene climate change in generating diversity in the Amazon region. Instead of restricting the role that allopatry has played only to inhabitants of wet, lowland forests, it seems likely that inhabitants of all Amazonian habitats should be subject to distributional shifts that could generate population structure.

Indeed, the emerging picture of Amazonia during the Pleistocene, based on data from fossil pollen [15], [28], [29], [30], simulations of paleoclimate, paleohabitat, and species' paleodistributions [58], [89], and, increasingly, by genetic data from Amazonian species [23] all point toward a similar scenario: temperatures, precipitation and carbon dioxide levels were all lower than today, but forests nevertheless remained widespread, and therefore species restricted to forest habitats were not dissected in the way envisioned by Haffer and colleagues [13], [16], [17], [86]. Nevertheless, our results suggest that these climate changes, perhaps acting on top of effects from earlier events such as marine incursions, may have been sufficient to drive diversification in some Amazonian species.

Our results suggest that the role that climate change has played in the diversification of Amazonian species should be revisited, but that other mechanisms that may act in concert should also be considered. That climate change in general is linked to diversification processes is also suggested by a number of recent studies that span various taxa, time periods and geographic regions [63], [90], [91], [92], [93], [94], [95]. The relationship between climate change and diversity is of particular interest for predicting the biotic effects of future climate change [96], [97], [98], [99], [100]. Combining paleodistribution modeling with comparative, molecular phylogeography across a diversity of taxa is likely to provide a productive framework for future research into this area.

## Materials and Methods

### Collection of samples and molecular analyses

Samples for molecular analysis were obtained from 118 *Atta cephalotes* colonies, 46 *Atta sexdens* colonies, and 30 *Atta laevigata* colonies, spanning the known geographic range of each species (Table S7). Sampling locations were chosen to allow testing of the hypotheses in question and to maximize coverage within each species' geographic range. Individual worker ants were collected at nests or along foraging trails and preserved in 95% ethanol during transport to The University of Texas at Austin (for samples collected outside Brazil) or São Paulo State University (UNESP), Rio Claro, SP, Brazil (for samples collected in Brazil), where they were stored at 4°C. The location of all samples was recorded using a handheld GPS unit (Garmin eTrex).

Two disjunct sections of mitochondrial DNA, encompassing part of the Cytochrome Oxidase I (COI) and tRNA-Leucine (tRNAleu) genes, as well as the entire intergenic spacer between COI and tRNAleu were sequenced for all samples. The sequences were concatenated into a single alignment for each species that varied in length from 635 base pairs in *A. cephalotes* to 701 base pairs in *A. sexdens* and *A. laevigata*. Several nuclear pseudogenes were accidentally amplified and sequenced for *A. cephalotes* (described in [101]) and were not used in subsequent analyses; all sequences included in the final alignments for each species appeared to be functional, mitochondrial loci, as no premature stop codons or frameshift mutations were detected. Additional sequences for outgroup taxa (*Atta columbica*, *Atta mexicana*, and *Atta texana*; see Table S7), used for phylogenetic analyses of *A. cephalotes* were obtained from specimens available in the Mueller Lab at The University of Texas at Austin. Sequence information for all samples was deposited in GenBank (Accession Numbers EU847821-EU848214).

Total genomic DNA was extracted from one individual per colony using either the DNeasy Blood and Tissue Kit (QIAGEN) or the AccuPrep Genomic DNA Extraction Kit (Bioneer, Inc.). Several sets of mtDNA primers (Table S1 and References S1) were used to amplify two sections of the cytochrome oxidase I (COI) gene, as well as an intergenic spacer, and a portion of the tRNA-Leucine gene. PCR reactions contained 1 ul each of genomic DNA (approximately 10 ng), 1X reaction buffer, dNTPs, and MgCl_2_, 0.04 ul of Taq polymerase, and 5.96 ul of water for a total reaction volume of 10 ul. Average PCR conditions were as follows, with slight modifications depending on the annealing temperatures of individual primer pairs: Initial denaturation at 95°C for 3 minutes was followed by 35 cycles of 95°C for 5 seconds, and an annealing temperature that increased by 0.5°C for each successive round of amplification, beginning at 45°C, for 20 seconds each round, with a final elongation step of 68°C for 15 seconds. PCR products were analyzed by running 3 ul of the product on a 1.5% agarose gel and subsequently visualized with ethidium bromide staining. For samples that successfully amplified, the remaining 7 ul of PCR product were purified by polyethylene glycol (PEG) precipitation, using a 1:1 PCR product/20% PEG mixture which was incubated for 15 min at 37°C followed by a 10-min centrifugation at 2,688×g and two washes with 80% ethanol.

Cycle sequencing reactions were performed for both forward and reverse sequences using the ABI BigDye Terminator Kit (version 3.1). Sephadex column purification was used to clean the cycle-sequencing product, which was then analyzed on a PRISM 3100 genetic analyzer (Applied Biosystems). Forward and reverse sequences were assembled into individual contigs using SeqMan II v.5.05 (DNASTAR) and alignments between sequences were created initially using Clustal X [102] and then adjusted manually in MacClade v. 4.06 [103].

### Paleodistribution modeling

Estimates of the current and historical potential geographic ranges of each species were made using Maxent version 2.3 [104]. Maxent uses presence-only species occurrence records (i.e. latitudes & longitudes of known species sightings) and environmental data (i.e. GIS layers) as input. In general, the maxent approach seeks to estimate an unknown (“target”) distribution using incomplete information about the target distribution and a given set of constraints. For modeling species potential geographical ranges, the occurrence data are considered to be the incomplete sample of a larger, unknown geographical distribution, and the environmental data are used as constraints [104], [105]. A recent comparison of methods for niche-based modeling of species potential ranges under current conditions identified Maxent as among the best approaches available in terms of predictive performance [56].

For each species, a model was constructed for the current potential range using known collection localities (see below) and current climate conditions; the model was then projected onto a reconstruction of climate layers for the LGM to obtain a potential geographic range of each species at the LGM. Localities used as known presence records for each species of leafcutter ant (Table S7) came primarily from observations by the authors. Additional localities were obtained from A. Himler, N. Gerardo, C. Currie, A. Little, A. Mikheyev, and S. Villamarin. Geographic coordinates for each locality were obtained using a handheld GPS unit (Garmin eTrex). Museum specimens, although abundant for many species of *Atta*, were generally not used in these analyses because they often do not contain detailed geographic coordinates indicating where the collection was made.

For current environmental conditions, twenty bioclimatic layers for the entire New World were obtained from the WorldClim dataset (http://www.worldclim.org; version 1.4), each with a resolution of approximately 10 km. The methods used to generate these layers are described in Hijmans et al. [108]. The “auto features” option was selected in Maxent for all analyses. In addition, the following settings were used for the full training runs for each species: 500 maximum iterations, a convergence threshold of 1.0E-5, “minimize memory use,” and a regularization multiplier equal to 1.0 [104].

Two approaches were used to determine whether the predictions for current conditions generated by Maxent were better than random predictions. First, the area under the receiver Operating Characteristic curve (AUC), a commonly used measurement for comparison of model performance [56], was calculated for each species. The AUC varies from 0 to 1, with greater scores indicating better discrimination ability; an AUC greater than 0.5 indicates that the model discriminates better than random [56].

Second, a separate analysis was conducted by randomly splitting the localities into two sets: training and testing. The training set (75% of localities for *A. cephalotes*, 90% for *A. laevigata* and *A. sexdens*) was used to build the model while the testing set was used to test the predictive ability of that model. The number of localities used for testing versus training was dependent on how many sites were available for each species. To test the predictive ability of the model, Maxent's cumulative prediction was converted to a binary (i.e. presence vs. absence) prediction. Ten different thresholds automatically generated by Maxent were used for this conversion and the extrinsic omission rate (the fraction of test localities that are outside the area in which the species is predicted to occur) was tested against the null hypothesis that it is no better than a random prediction (of equal area) using a one-tailed binomial test [104]. The same settings were used as for the full training runs, except that all of the available samples were used to build the model.

Estimates of the potential geographic range of each species during the last glacial maximum (LGM, approximately 21 kya) were made by projecting the model generated under current environmental conditions onto a reconstruction of the same environmental variables at the LGM (see [57] for an explanation of how these layers were generated). A binary (presence vs. absence) prediction for the LGM was necessary for hypothesis tests (see below). To obtain a binary prediction, threshold values were chosen that minimized the commission (false positive) rate for current conditions, based on absence data obtained from recent surveys (S. Solomon, unpublished). The cumulative probability thresholds chosen for *A. cephalotes*, *A. sexdens*, and *A. laevigata* were 1, 5, and 5, respectively. The results of the paleodistribution models were used in subsequent analyses to provide a priori population groupings for all tests of the refugia hypothesis in the following way: areas that were predicted to provide contiguous blocks of suitable habitat during the LGM (using the binary prediction) were grouped together as a single population (Figure 1: C, F, I); areas that were predicted not to be suitable were ignored for the purposes of hypothesis testing (see below).

### Gene tree topology tests

Each hypothesis makes a specific prediction about the genealogical relationships between populations across the geographic range of each species (see Table 1). Specifically, given enough time, isolated populations that have diverged evolutionarily are expected to become reciprocally monophyletic [14], [87]. The relationships predicted by a strict interpretation of each hypothesis, assuming complete lineage sorting, were converted into backbone constraint topologies as follows. For the riverine barrier hypothesis, populations occurring on either bank (i.e. north and south) of the Amazon River should be reciprocally monophyletic. For the marine incursion hypothesis, populations near the eastern base of the Andes, on the Brazilian Shield, and on the Guyana Shield should be reciprocally monophyletic. For the refugia hypothesis, populations that were predicted by the paleodistribution models to persist during the last glacial maximum (Figure 1, middle rows) should be reciprocally monophyletic.

To determine whether these predictions were met, mitochondrial DNA gene trees were estimated, using unique haplotypes, with maximum likelihood and Bayesian inference techniques. Maximum likelihood searches were performed with a beta version of GARLI [109] that allows backbone constraints (version 0.952 Beta), with default settings and parameters estimated according to the model of evolution selected using the Akaike Information Criterion (AIC) as implemented in ModelTest [110]. The best tree consistent with the constraint topology for each hypothesis was then found using identical settings. In order to assess whether the null hypothesis represented by the constraint trees could be rejected, the difference between the log-likelihood values of the best constrained and best unconstrained trees was used as a test statistic, with statistical significance assessed through simulation (parametric bootstrap or SOWH test [111], [112]). One hundred simulated datasets were generated using Seq-gen [113], with parameters estimated by PAUP* [114] from the best constrained tree under each constraint. Constrained and unconstrained searches were performed in GARLI on the simulated data using identical settings as for the empirical data. The distribution of differences between constrained and unconstrained searches on the simulated data was used to assess the significance of the test statistic; the *p* value was equal to the number of simulated datasets (out of 100 replicates) with a difference in log-likelihood scores between constrained and unconstrained searches greater than the empirical difference. The null hypothesis (i.e. constraint topology) was rejected when *p* values were less than 0.05.

Bayesian searches were conducted in MrBayes version 3.1.2 [115]. Four separate runs were conducted, each with four incrementally heated chains and uninformative, default priors; convergence and optimal burn-in were assessed as described in [116] using the program MrConverge (A. Lemmon, in prep.). After discarding burn-in, the posterior samples of tree topologies for each run were combined in PAUP*; the combined posterior sample was then filtered with the constraint tree for each hypothesis. The proportion of trees retained by the filter was the Bayesian posterior probability of that hypothesis.

### Population Genetic Structure

To determine whether populations are structured as predicted by each of the hypotheses in question (Table 1), two types of population-genetic analyses were performed, using all ingroup haplotypes for each. Analysis of molecular variance (AMOVA) was used, as implemented in Arlequin 3.11 [117], to calculate the percentage of variance explained by *a priori* population groupings in a hierarchical framework [118]. The population structure was defined for each species/hypothesis, as for the constraint trees in phylogenetic analyses. Tamura and Nei distances with an alpha shape parameter were used to compute the pairwise distance matrix for all AMOVA calculations, as this is the most complex model of sequence evolution currently available in Arlequin [117]. Transitions and transversions were given equal weight, while deletions (i.e. gaps) were ignored. Statistical significance of variance components was assessed using the permutation procedures described in the Arlequin user's manual (http://cmpg.unibe.ch/software/ arlequin3/arlequin31.pdf).

To further test for the presence of barriers to gene flow, as predicted for each hypothesis, simple and partial Mantel tests [119], [120] were conducted on the following matrices. First, the pairwise maximum likelihood genetic distance between individuals (as defined *a priori* for each species/hypothesis) was calculated in PAUP, using the model of sequence evolution selected by the AIC in ModelTest [110]. Second, the pairwise geographic distance (in kilometers) was calculated using the program Range (http://earthquake.usgs.gov/research/ software/#Range). Third, the presence or absence of a potential barrier between two individuals was coded as a binary character and converted to a pairwise barrier matrix. If the straight-line distance between two individuals crossed the barrier of interest (e.g. the Amazon River in the case of the riverine barrier hypothesis), then the barrier was coded as present; if not, the barrier was coded as absent.

For each hypothesis/species, simple Mantel tests assessed the correlation between pairwise genetic distance matrices and the pairwise barrier matrix. Furthermore, isolation by distance was tested for by a simple Mantel test of the pairwise genetic distance and pairwise geographic distance. If both of the above tests were statistically significant, a partial Mantel test was conducted to determine whether the genetic distance between individuals was correlated with the presence of a potential barrier when the effects of geographic distance are removed. All Mantel tests were conducted with the program zt [121] and used 10,000 permutations to assess statistical significance.

### Demographic Analyses

Two types of analyses were performed using Arlequin 3.11 [117] to test the predictions of both the refugia and marine incursion hypotheses that populations restricted to an isolated region should show signs of population bottlenecks and subsequent population expansion (Table 1). Tajima's *D* statistic [122] which is expected to be negative for populations that have experienced recent population growth [123], was calculated for each population grouping (Figure 1) for each hypothesis. Significance was tested, as described in the Arlequin manual (http://cmpg.unibe.ch/ software/arlequin3/arlequin31.pdf) by simulating random samples under a model of population equilibrium, where the p value is equal to the number of simulated values less than or equal to the observed value of *D*.

Second, pairwise nucleotide mismatch distributions were calculated for each population. A population that is at equilibrium is expected to have a multi-model mismatch distribution due to the stochastic shape of its gene tree, whereas populations that have experienced recent growth should have a unimodal mismatch distribution resulting from a star-like gene tree [124], [125]. A model of stepwise population expansion was estimated using a generalized least-square approach [126], and its validity was tested as follows: The sum of squared deviations (SSD) between the observed and the simulated (i.e. expected) mismatch distributions was used as a test statistic; 1000 bootstrap simulations of the data were performed, and the SSD was calculated for each; the null hypothesis of population expansion was rejected when fewer than 5% of the simulated SSD values were greater than the observed SSD. To further test whether the observed mismatch distributions deviated from the null expectations characteristic of an expanding population, Harpending's Raggedness Index [127] was calculated. This index has greater values for distributions that are multimodal, as expected for stationary (i.e. non-expanding) populations. Significance for Harpending's Raggedness Index was assessed through bootstrap simulation as described for the SSD.

### Coalescent dating of population divergence

The refugia and marine incursion hypotheses make similar predictions about how populations should be structured (see Table 1). However, these two hypotheses make predictions on vastly different temporal scales. On the one hand, the Pleistocene refugia model predicts that current population structure formed during or subsequent to the Pleistocene, 10,000 to 1.8 million years ago. In contrast, the population structure predicted by the marine incursion hypothesis should date to the Miocene, approximately 10–15 million years ago.

To discriminate between these alternative scenarios, a coalescent dating approach was used. The results of the phylogenetic analyses for each species were used to determine where the most basal split occurred between all sampled populations. The approximate date of this split, in years before present (ybp), was estimated using the isolation-with-migration model developed by Nielsen and Wakeley [128] as implemented in the program IM [129]. This program simultaneously approximates the divergence time (t) between two populations that share a common ancestor, the migration rates (m_1_ and m_2_) between these populations, the proportion of the ancestral population that founded each of the resulting populations (*s* and 1-*s*) and a measure of genetic diversity for the ancestral (theta_A_) as well as both resulting populations (theta_1_, theta_2_) in a Bayesian framework using a Markov chain Monte Carlo method. The program assumes that the diverging populations are not exchanging migrants with any other populations [128].

Preliminary analyses were conducted on each population pair to assess mixing of the chains, as well as to determine appropriate priors for the parameters that were not of interest (i.e. all but t; see Table S6 for a list of the priors used for each species). The upper limit for the prior distribution of t, t_max_, was determined based on recent estimates for the origin of the genus *Atta* [81]; the oldest possible date recovered by that study for the origin of the crown group of leafcutter ants, 14 mya (Schultz and Brady 2008 Table S3), was used as t_max_ for all three species in our study. All searches used the HKY model of sequence evolution (currently the most appropriate model available in IM for mtDNA evolution), a generation time of 4 years (based on life history data from Autuori [130] and observations by the authors) and uninformative priors. After the first 100,000 steps, which were discarded as burnin, searches proceeded until the following criteria were satisfied: (1) the minimum ESS was at least 100, (2) no trends were observable in plots of parameter values throughout the course of the run, and (3) the results from at least 3 independent runs using the same data and prior values converged on similar posterior distributions.

The estimates for t were converted into time in years since divergence (T_div_) using the equation, T_div_ = t*u, where *u* is the mutation rate in substitutions per site per year. The mutation rate for COI was estimated based on unpublished sequence data for the same gene from species spanning the tribe Attini and from divergence times within the Attini, as reconstructed by Schultz and Brady [81]; the resulting value of 9.5 substitutions per site per million years is consistent with an estimate of the average mutation rate for COI in a recent survey across the arthropods [131].

## Supporting Information

Table S1 Mitochondrial DNA primers used for amplification and sequencing of ants in the present study.
(0.05 MB DOC)

Table S2 Results of gene tree topology tests. For the parametric bootstrap analyses, p values less than 0.05 indicate rejection of the null hypothesis (i.e. the constraint tree). Bpp is the Bayesian posterior probability of a given constraint topology (*The predictions of the Pleistocene refugia and marine incursion hypotheses are identical for A. laevigata).
(0.07 MB DOC)

Table S3 Results of Analyses of Molecular Variance (AMOVA). For each hypothesis, population structure was defined as predicted by each hypothesis (see text). The percentage of variance explained by each hierarchical grouping is shown, with an asterix indicating statistical significance as assessed by permutation. The “among regions” grouping is the grouping of interest for the purposes of hypothesis testing in this study ( Negative percentages and percentages greater than 100 should be interpreted as not significantly different than zero and 100, respectively).
(0.07 MB DOC)

Table S4 Results of simple and partial Mantel tests of matrix correlation. For each hypothesis, the correlation between corrected, pairwise genetic distance between individuals and the presence or absence of the barrier of interest was tested using a simple Mantel test (Gen Dist×Barrier). The correlation between genetic and geographic distances (Gen Dist×Geog Dist) was assessed to test for isolation by distance. If a significant correlation was found between both matrix comparisons, a partial Mantel test was conducted on all three matrices to determine whether the presence of the barrier of interest was significantly correlated with genetic distance once the effects of geographic distance are factored out (Partial). All tests used 10,000 permutations to assess statistical significance.
(0.06 MB DOC)

Table S5 Results of demographic analyses. Pairwise nucleotide mismatch distributions and Tajima's (1989) D tests were used to test for historical population expansion for populations defined a priori for each hypothesis.
(0.08 MB DOC)

Table S6 Summary of coalescent dating analyses using the program IM. Left panel: priors used for estimating Tdiv, the time since earliest population divergence for each species. Right panel: the posterior estimate for Tdiv, as well as the lower (95Lo) and upper (95Hi) 95% confidence limits for each species.
(0.04 MB DOC)

Table S7 List of all samples used, their geographic locations, and GenBank Accession numbers for samples used in molecular analyses. (BR = Brazil; BZ = Belize; CO = Colombia; CR = Costa Rica; EC = Ecuador; FG = French Guiana; GT = Guatemala; GU = Guyana; MX = Mexico; PA = Panama; PU = Peru; TR = Trinidad; US = United States; VZ = Venezuela)
(0.44 MB DOC)

References S1 (0.02 MB DOC)
