# Supplementary material for: Paleodistributions and Comparative Molecular Phylogeography of Leafcutter Ants (Atta spp.) Provide New Insight into the Origins of Amazonian Diversity
Source: PLoS One. 2008 Jul 23;3(7):e2738. doi: 10.1371/journal.pone.0002738 (PMC2447876; doi:10.1371/journal.pone.0002738)
Supplement: Table S1 — Mitochondrial DNA primers used for amplification and sequencing of ants in the present study. (0.05 MB DOC) [file pone.0002738.s001.doc]

| Primer Name | Region amplified | Forward/Reverse | Primer Sequence (5'-3') | Reference |
| --- | --- | --- | --- | --- |
| Jerry | COI | F | CAACATTTATTTTGATTTTTTGG | [132] |
| George I | COI-COII | F | ATACCTCGACGTTATTCAGA | [133] |
| AntF | COI-COII | F | ATTCATTCTTATCTTGAAATATTATTTC | [101] |
| Ben | COI | R | GCTACTACATAATAKGTATCATG | [S1] |
| AntR | COI-COII | R | TTCATAAGTTCAGTATCATTGGTG | [101] |
| SESR1EXT1 | COI-COII | R | ATTATTAAGTCGTATGTAGGGGA | This study |
| ASMtRNAleuR1 | COI-COII | R | CAATGCACTATTCTGCCATATTAAA | This study |

Table S1: Mitochondrial DNA primers used for amplification and sequencing of ants in the present study.
