# Supplementary material for: Paleodistributions and Comparative Molecular Phylogeography of Leafcutter Ants (Atta spp.) Provide New Insight into the Origins of Amazonian Diversity
Source: PLoS One. 2008 Jul 23;3(7):e2738. doi: 10.1371/journal.pone.0002738 (PMC2447876; doi:10.1371/journal.pone.0002738)
Supplement: Table S2 — Results of gene tree topology tests. For the parametric bootstrap analyses, p values less than 0.05 indicate rejection of the null hypothesis (i.e. the constraint tree). Bpp is the Bayesian posterior probability of a given constraint topology (*The predictions of the Pleistocene refugia and marine incursion hypotheses are identical for A. laevigata). (0.07 MB DOC) [file pone.0002738.s002.doc]

|  | Parametric Bootstrap Tests | | | | Bayesian Tests | | |
| --- | --- | --- | --- | --- | --- | --- | --- |
| Constraint Name | -ln L constrained | -ln L uncon-strained | Difference | *p* | # consistent trees | # trees in posterior sample | Bpp |
| *Atta cephalotes* |  |  |  |  |  |  |  |
| Amazon River | 2263.5432 | 2195.4377 | 68.1055 | <0.001 | 0 | 223872 | 0 |
| Marine Incursion | 2266.8397 | 2195.4377 | 71.4019 | <0.001 | 0 | 223872 | 0 |
| Pleistocene Refugia | 2270.3737 | 2195.4377 | 74.936 | <0.001 | 0 | 223872 | 0 |
| *Atta sexdens* |  |  |  |  |  |  |  |
| Amazon River | 2159.8489 | 2096.8147 | 63.0342 | <0.001 | 0 | 11832 | 0 |
| Marine Incursion | 2104.9211 | 2096.8147 | 8.10643 | <0.001 | 0 | 11832 | 0 |
| Pleistocene Refugia | 2096.8836 | 2096.8147 | 0.0689 | 0.15 | 9975 | 11832 | 0.843 |
| *Atta laevigata* |  |  |  |  |  |  |  |
| Amazon River | 1965.9731 | 1933.9096 | 32.0635 | <0.001 | 0 | 7812 | 0 |
| Refugia/Marine^*^ | 1974.7753 | 1933.9096 | 40.8657 | <0.001 | 0 | 7812 | 0 |

Table S2: Results of gene tree topology tests. For the parametric bootstrap analyses, *p* values less than 0.05 indicate rejection of the null hypothesis (i.e. the constraint tree). Bpp is the Bayesian posterior probability of a given constraint topology (*The predictions of the Pleistocene refugia and marine incursion hypotheses are identical for *A. laevigata*).
