# Supplementary material for: Paleodistributions and Comparative Molecular Phylogeography of Leafcutter Ants (Atta spp.) Provide New Insight into the Origins of Amazonian Diversity
Source: PLoS One. 2008 Jul 23;3(7):e2738. doi: 10.1371/journal.pone.0002738 (PMC2447876; doi:10.1371/journal.pone.0002738)
Supplement: Table S3 — Results of Analyses of Molecular Variance (AMOVA). For each hypothesis, population structure was defined as predicted by each hypothesis (see text). The percentage of variance explained by each hierarchical grouping is shown, with an asterix indicating statistical significance as assessed by permutation. The “among regions” grouping is the grouping of interest for the purposes of hypothesis testing in this study ( Negative percentages and percentages greater than 100 should be interpreted as not significantly different than zero and 100, respectively). (0.07 MB DOC) [file pone.0002738.s003.doc]

| Species | Hypothesis | N | Among regions | Among populations within regions | Within populations |
| --- | --- | --- | --- | --- | --- |
| *A. cephalotes* | Amazon River | 20 | -7.81[[1]](#footnote-2) | 54.41* | 53.40* |
|  | Marine Incursion | 47 | 30.24 | 48.96* | 20.79* |
|  | Pleistocene Refugia | 82 | 40.19* | 33.40* | 26.41* |
| *A. sexdens* | Amazon River | 17 | -48.91 | 119.87* | 29.04* |
|  | Marine Incursion | 24 | 20.3 | 74.13* | 5.57* |
|  | Pleistocene Refugia | 30 | 39.42 | 56.06* | 4.52* |
| *A. laevigata* | Amazon River | 13 | -98.56 | 186.84* | 11.72* |
|  | Marine Incursion/ Refugia | 18 | 59.24 | -0.36 | 41.12* |

Table S3: Results of Analyses of Molecular Variance (AMOVA). For each hypothesis, population structure was defined as predicted by each hypothesis (see text). The percentage of variance explained by each hierarchical grouping is shown, with an asterix indicating statistical significance as assessed by permutation. The “among regions” grouping is the grouping of interest for the purposes of hypothesis testing in this study

( Negative percentages and percentages greater than 100 should be interpreted as not significantly different than zero and 100, respectively).

1. [↑](#footnote-ref-2)
