# Supplementary material for: Paleodistributions and Comparative Molecular Phylogeography of Leafcutter Ants (Atta spp.) Provide New Insight into the Origins of Amazonian Diversity
Source: PLoS One. 2008 Jul 23;3(7):e2738. doi: 10.1371/journal.pone.0002738 (PMC2447876; doi:10.1371/journal.pone.0002738)
Supplement: Table S4 — Results of simple and partial Mantel tests of matrix correlation. For each hypothesis, the correlation between corrected, pairwise genetic distance between individuals and the presence or absence of the barrier of interest was tested using a simple Mantel test (Gen Dist×Barrier). The correlation between genetic and geographic distances (Gen Dist×Geog Dist) was assessed to test for isolation by distance. If a significant correlation was found between both matrix comparisons, a partial Mantel test was conducted on all three matrices to determine whether the presence of the barrier of interest was significantly correlated with genetic distance once the effects of geographic distance are factored out (Partial). All tests used 10,000 permutations to assess statistical significance. (0.06 MB DOC) [file pone.0002738.s004.doc]

|  |  | Gen Dist x Barrier | | Gen Dist x Geog Dist | | Partial | |
| --- | --- | --- | --- | --- | --- | --- | --- |
| Species | Hypothesis | *r* | *p* | *r* | *p* | *r* | *p* |
| *A. cephalotes* | Riverine barrier | 0.113 | 0.12714 | 0.729 | 0.0001 | N/A | N/A |
|  | Marine incursion | 0.265 | 0.00002 | 0.665 | 0.00001 | -0.149 | 0.00003 |
|  | Pleistocene refugia | 0.364 | 0.00001 | 0.498 | 0.00001 | 0.076 | 0.00589 |
| *A. sexdens* | Riverine barrier | 0.119 | 0.07756 | 0.801 | 0.00001 | N/A | N/A |
|  | Marine incursion | 0.561 | 0.00014 | 0.785 | 0.00001 | -0.396 | 0.00138 |
|  | Pleistocene refugia | 0.593 | 0.00001 | 0.574 | 0.00001 | 0.251 | 0.00009 |
| *A. laevigata* | Riverine barrier | 0.024157 | 0.355466 | 0.707757 | 0.00128 | N/A | N/A |
|  | Marine/ Refugia | 0.580717 | 0.00977 | 0.383795 | 0.02435 | 0.472043 | 0.0073 |

Table S4: Results of simple and partial Mantel tests of matrix correlation. For each hypothesis, the correlation between corrected, pairwise genetic distance between individuals and the presence or absence of the barrier of interest was tested using a simple Mantel test (Gen Dist x Barrier). The correlation between genetic and geographic distances (Gen Dist x Geog Dist) was assessed to test for isolation by distance. If a significant correlation was found between both matrix comparisons, a partial Mantel test was conducted on all three matrices to determine whether the presence of the barrier of interest was significantly correlated with genetic distance once the effects of geographic distance are factored out (Partial). All tests used 10,000 permutations to assess statistical significance.
