# Supplementary material for: Paleodistributions and Comparative Molecular Phylogeography of Leafcutter Ants (Atta spp.) Provide New Insight into the Origins of Amazonian Diversity
Source: PLoS One. 2008 Jul 23;3(7):e2738. doi: 10.1371/journal.pone.0002738 (PMC2447876; doi:10.1371/journal.pone.0002738)
Supplement: Table S5 — Results of demographic analyses. Pairwise nucleotide mismatch distributions and Tajima's (1989) D tests were used to test for historical population expansion for populations defined a priori for each hypothesis. (0.08 MB DOC) [file pone.0002738.s005.doc]

|  |  |  | Mismatch Distribution | | | | Tajima’s *D* | |
| --- | --- | --- | --- | --- | --- | --- | --- | --- |
| Species | Hypothesis | Population | SSD | *p* | Raggedness | *p* | *D* | *p* |
| *A. cephalotes* | Marine incursion | Andes | 0.00854517 | 0.428 | 0.08645395 | 0.315 | -1.1544 | 0.142 |
|  |  | Guiana Shield | 0.03722933 | 0.231 | 0.0538843 | 0.592 | 0.25014 | 0.639 |
|  |  | Brazilian Shield | 0.04740862 | 0.092 | 0.02787182 | 0.589 | 0.79628 | 0.827 |
|  | Pleistocene refugia | Atlantic Coast | 0.0200829 | 0.299 | 0.08930211 | 0.3 | -1.65893 | 0.033 |
|  |  | Greater Amazonia | 0.02135842 | 0.679 | 0.02513078 | 0.547 | -0.59127 | 0.309 |
|  |  | S Central America | 0.24169597 | 0.004 | 0.01579238 | 1 | -1.85767 | 0.007 |
|  |  | N Central America | 0.08286126 | 0.309 | 0.14 | 0.845 | 1.21852 | 0.864 |
| *A. sexdens* | Marine incursion | Brazilian Shield | 0.20368588 | 0.137 | 0.47 | 0.191 | -1.21852 | 0.026 |
|  |  | Guiana Shield | 0.36626913 | 0 | 0.21440472 | 0.98 | -0.13367 | 0.483 |
|  |  | Andes | N/A | N/A | N/A | N/A | N/A | N/A |
|  | Pleistocene refugia | Atlantic Coast | 0.4999998 | 0 | 0.75 | 0.966 | -0.78012 | 0.198 |
|  |  | Western Amazon | 0.32561711 | 0 | 0.59027778 | 0.931 | -1.03227 | 0.222 |
|  |  | Guiana  Shield | 0.31271602 | 0.003 | 0.25859645 | 0.972 | 0.0198 | 0.577 |
| *A. laevigata* | Marine Incursion/ Refugia | Guiana Shield | 0.01959799 | 0.181 | 0.10577614 | 0.212 | -2.31554 | 0 |
|  |  | Brazilian Shield | N/A | N/A | N/A | N/A | 0 | 1 |

Table S5: Results of demographic analyses. Pairwise nucleotide mismatch distributions and Tajima’s (1989) *D* tests were used to test for historical population expansion for populations defined a priori for each hypothesis.
