# Supplementary material for: Paleodistributions and Comparative Molecular Phylogeography of Leafcutter Ants (Atta spp.) Provide New Insight into the Origins of Amazonian Diversity
Source: PLoS One. 2008 Jul 23;3(7):e2738. doi: 10.1371/journal.pone.0002738 (PMC2447876; doi:10.1371/journal.pone.0002738)
Supplement: Table S6 — Summary of coalescent dating analyses using the program IM. Left panel: priors used for estimating Tdiv, the time since earliest population divergence for each species. Right panel: the posterior estimate for Tdiv, as well as the lower (95Lo) and upper (95Hi) 95% confidence limits for each species. (0.04 MB DOC) [file pone.0002738.s006.doc]

|  | Priors | | | | | | Posterior Estimates | | |
| --- | --- | --- | --- | --- | --- | --- | --- | --- | --- |
| Species | q1 | q2 | qA | m1 | m2 | t_max_ | T_div_ | 95_Lo_ | 95_Hi_ |
| *A. cephalotes* | 10.5 | 1049.65 | 1049.65 | 1 | 1 | 133 | 1,421,000 | 819,000 | 4,893,000 |
| *A. sexdens* | 120.59 | 120.59 | 120.59 | 1 | 1 | 133 | 1,099,000 | 987,000 | 13,279,000 |
| *A. laevigata* | 12.12 | 1211.62 | 1211.62 | 1 | 1 | 133 | 609,000 | 371,000 | 12,817,000 |

Table S6: Summary of coalescent dating analyses using the program IM. *Left panel*: priors used for estimating T_div_, the time since earliest population divergence for each species. *Right panel*: the posterior estimate for T_div_, as well as the lower (95_Lo_) and upper (95_Hi_) 95% confidence limits for each species.
