# Supplementary material for: Paleodistributions and Comparative Molecular Phylogeography of Leafcutter Ants (Atta spp.) Provide New Insight into the Origins of Amazonian Diversity
Source: PLoS One. 2008 Jul 23;3(7):e2738. doi: 10.1371/journal.pone.0002738 (PMC2447876; doi:10.1371/journal.pone.0002738)
Supplement: Table S7 — List of all samples used, their geographic locations, and GenBank Accession numbers for samples used in molecular analyses. (BR = Brazil; BZ = Belize; CO = Colombia; CR = Costa Rica; EC = Ecuador; FG = French Guiana; GT = Guatemala; GU = Guyana; MX = Mexico; PA = Panama; PU = Peru; TR = Trinidad; US = United States; VZ = Venezuela) (0.44 MB DOC) [file pone.0002738.s007.doc]

| **Species** | **ID** | **Latitude** | **Longitude** | **Country: Site** | **GenBank Accession #** |
| --- | --- | --- | --- | --- | --- |
| *A. cephalotes* | AGH03061101 | -0.497516667 | -76.37471667 | EC: La Selva | EU847828,EU847949 |
| *A. cephalotes* | AGH030612-01 | -0.497516667 | -76.37471667 | EC: La Selva | EU847829,EU847950 |
| *A. cephalotes* | AL030612-02 | -0.497516667 | -76.37471667 | EC: La Selva | EU847830,EU847951 |
| *A. cephalotes* | AL030614-02 | -0.63825 | -76.14931667 | EC: Tiputini | EU847831,EU847952 |
| *A. cephalotes* | Alenquer 3 | -1.91676 | -54.62662 | BR: Alenquer 3 |  |
| *A. cephalotes* | Almirante 1 | 9.14857 | -82.32436 | PA: Pta Peña-Almirante 1 |  |
| *A. cephalotes* | Almirante 3 | 9.04552 | -82.30249 | PA: Pta Peña-Almirante 3 |  |
| *A. cephalotes* | Alta Floresta 2 | -9.59214 | -56.02422 | BR: Alta Floresta 2 |  |
| *A. cephalotes* | Alta Floresta 3 | -9.65121 | -56.01799 | BR: Alta Floresta 3 |  |
| *A. cephalotes* | Alta Floresta 5 | -9.53129 | -55.99617 | BR: Alta Floresta 5 |  |
| *A. cephalotes* | ATMCAVE | 17.216666 | -88.833333 | BZ: ATM Cave | EU847832,EU847953 |
| *A. cephalotes* | BCI | 9.16425 | -79.84784 | PA: BCI |  |
| *A. cephalotes* | Cahuita 2 | 9.735816667 | -82.83786667 | CR: Cahuita 2 |  |
| *A. cephalotes* | Cahuita 3 | 9.7343 | -82.82808333 | CR: Cahuita 3 |  |
| *A. cephalotes* | Calderas 1 | 8.83354 | -70.4947 | VZ: Calderas 1 |  |
| *A. cephalotes* | Calderas 3 | 8.85996 | -70.47611 | VZ: Calderas 3 |  |
| *A. cephalotes* | Calderas 4 | 8.89603 | -70.44917 | VZ: Calderas 4 |  |
| *A. cephalotes* | Calderas 5 | 8.91012 | -70.44672 | VZ: Calderas 5 |  |
| *A. cephalotes* | Canaima | 6.2465 | -62.85368 | VZ: Canaima |  |
| *A. cephalotes* | Changuinola 1 | 9.33111 | -82.46096 | PA: AlmiranteChanguinola1 |  |
| *A. cephalotes* | CINCIZOO | 10.683611 | -61.283333 | TR: ? | EU847833,EU847954 |
| *A. cephalotes* | CO-650 | 6.468333 | -75.035555 | CO: San Roque | EU847821,EU847942 |
| *A. cephalotes* | CO-651 | 6.469166 | -75.034722 | CO: San Roque | EU847822,EU847943 |
| *A. cephalotes* | CO-688 | 6.534722 | -75.884444 | CO: Santafé de Antioquia | EU847823,EU847944 |
| *A. cephalotes* | CO-692 | 6.533333 | -75.883333 | CO: Santafé de Antioquia | EU847824,EU847945 |
| *A. cephalotes* | CO-693 | 6.535277 | -75.883888 | CO: Santafé de Antioquia | EU847825,EU847946 |
| *A. cephalotes* | CO-697 | 5.667222 | -75.850833 | CO: Andes | EU847826,EU847947 |
| *A. cephalotes* | CO-699 | 5.97 | -75.72 | CO: Venecia | EU847827,EU847948 |
| *A. cephalotes* | Coclesito | 8.68655 | -80.4557 | PA: Coclesito |  |
| *A. cephalotes* | E909 | -14.201777 | -39.82375 | BR: Aurelino Leal | EU847834,EU847955 |
| *A. cephalotes* | E958 | -9.55657 | -55.997 | BR: Alta Floresta | EU847835,EU847956 |
| *A. cephalotes* | Farm | -14.20027778 | -39.81586111 | BR: Farm |  |
| *A. cephalotes* | Fort Sherman | 9.33388 | -79.79851 | PA: Fort Sherman |  |
| *A. cephalotes* | Ipiau | -14.09619444 | -39.78102778 | BR: Ipiau |  |
| *A. cephalotes* | Isla Colón | 9.39198 | -82.23982 | PA: Isla Colón |  |
| *A. cephalotes* | Isla Cristobal | 9.29051 | -82.26089 | PA: Isla Cristobal |  |
| *A. cephalotes* | KM-GU | 5.3 | -59.9 | GU: Unknown | EU847836,EU847957 |
| *A. cephalotes* | Macapa 2 | 0.60029 | -51.75318 | BR: Macapa 2 |  |
| *A. cephalotes* | Macapa 3 | 0.61917 | -51.70161 | BR: Macapa 3 |  |
| *A. cephalotes* | Monte Carmelo | 9.25677 | -70.85779 | VZ: Monte Carmelo |  |
| *A. cephalotes* | NMG030609-04 | -0.497516667 | -76.37471667 | EC: La Selva | EU847837,EU847958 |
| *A. cephalotes* | NMG030611-01 | -0.497516667 | -76.37471667 | EC: La Selva | EU847838,EU847959 |
| *A. cephalotes* | Pacuarito | 10.10153333 | -83.46098333 | CR: Pacuarito |  |
| *A. cephalotes* | Palenque1 | 17.49168333 | -92.02481667 | MX: Palenque1 |  |
| *A. cephalotes* | Palenque2 | 17.49153333 | -92.02375 | MX: Palenque2 |  |
| *A. cephalotes* | Palenque3 | 17.49 | -92.02366667 | MX: Palenque3 |  |
| *A. cephalotes* | Palenque4 | 17.49005 | -92.02361667 | MX: Palenque4 |  |
| *A. cephalotes* | Palenque5 | 17.51018333 | -91.98591667 | MX: Palenque5 |  |
| *A. cephalotes* | Pillcopata 1 | -13.03925 | -71.50857 | PU: Pillcopata 1 |  |
| *A. cephalotes* | Pillcopata 2 | -13.03933 | -71.50725 | PU: Pillcopata 2 |  |
| *A. cephalotes* | Pillcopata 3 | -13.02342 | -71.49075 | PU: Pillcopata 3 |  |
| *A. cephalotes* | Pillcopata 4 | -13.02591 | -71.48086 | PU: Pillcopata 4 |  |
| *A. cephalotes* | Pillcopata 5 | -13.02226 | -71.46418 | PU: Pillcopata 5 |  |
| *A. cephalotes* | Pipeline Road | 9.118769 | -79.7097 | PA: Pipeline Road |  |
| *A. cephalotes* | RIH-ESMEC | 0.403333 | -79.973333 | EC: Finca Tenorio-La Tola | EU847839,EU847960 |
| *A. cephalotes* | Rio Grande 2 | 8.4224 | -61.67191 | VZ: Camp Rio Grande 2 |  |
| *A. cephalotes* | Santerem | -2.55767 | -54.72733 | BR: Santerem |  |
| *A. cephalotes* | SES020106-01 | 9.652666667 | -82.75198333 | CR: Limon | EU847840,EU847961 |
| *A. cephalotes* | SES020107-01 | 9.638683333 | -82.6934 | CR: Pto Viejo Talamanca | EU847841,EU847962 |
| *A. cephalotes* | SES020107-02 | 9.6396 | -82.69281667 | CR: Pto Viejo Talamanca | EU847842,EU847963 |
| *A. cephalotes* | SES020107-05 | 9.736033333 | -82.83843333 | CR: Cahuita | EU847843,EU847964 |
| *A. cephalotes* | SES020107-07 | 9.73845 | -82.82213333 | CR: Cahuita | EU847844,EU847965 |
| *A. cephalotes* | SES020108-03 | 9.874966667 | -83.009 | CR: Banano del Sur | EU847845,EU847966 |
| *A. cephalotes* | SES020109-01 | 9.992483333 | -83.12618333 | CR: Moín | EU847846,EU847967 |
| *A. cephalotes* | SES020109-02 | 10.02683333 | -83.24896667 | CR: Matina-Limón | EU847847,EU847968 |
| *A. cephalotes* | SES020110-01 | 10.50718333 | -84.0309 | CR: Tres Rosales | EU847848,EU847969 |
| *A. cephalotes* | SES020113-01 | 10.4299 | -84.0097 | CR: La Selva Arboretum | EU847849,EU847970 |
| *A. cephalotes* | SES020113-04 | 10.42926667 | -84.00995 | CR: La Selva Arboretum | EU847850,EU847971 |
| *A. cephalotes* | SES020113-06 | 10.42951667 | -84.0106 | CR: La Selva Arboretum | EU847851,EU847972 |
| *A. cephalotes* | SES020113-07 | 10.42966667 | -84.01073333 | CR: La Selva Arboretum | EU847852,EU847973 |
| *A. cephalotes* | SES020113-10 | 10.43008333 | -84.01118333 | CR: La Selva Arboretum | EU847853,EU847974 |
| *A. cephalotes* | SES020524-14 | 9.32605 | -79.95731 | PA: Fort Sherman | EU847854,EU847975 |
| *A. cephalotes* | SES020531-04 | 8.99978 | -82.26275 | PA: Punta Peña-Almirante | EU847855,EU847976 |
| *A. cephalotes* | SES020602-05 | 9.02712 | -82.30523 | PA: Punta Peña-Almirante | EU847856,EU847977 |
| *A. cephalotes* | SES020603-02 | 9.29537 | -82.42819 | PA: Almirante-Changuinola | EU847857,EU847978 |
| *A. cephalotes* | SES020604-02 | 9.0982 | -82.2895 | PA: Punta Peña-Almirante | EU847858,EU847979 |
| *A. cephalotes* | SES020606-04 | 9.34018 | -82.17691 | PA: Isla Bastimentos | EU847859,EU847980 |
| *A. cephalotes* | SES020609-01 | 9.20105 | -82.13069 | PA: Isla Popa | EU847860,EU847981 |
| *A. cephalotes* | SES020609-02 | 9.20105 | -82.13069 | PA: Isla Popa | EU847861,EU847982 |
| *A. cephalotes* | SES020609-03 | 9.20105 | -82.13069 | PA: Isla Popa | EU847862,EU847983 |
| *A. cephalotes* | SES030112-02 | 18.48846667 | -95.06698333 | MX: Sierra de los Tuxtlas | EU847863,EU847984 |
| *A. cephalotes* | SES030112-04 | 18.48846667 | -95.06698333 | MX: Sierra de los Tuxtlas | EU847864,EU847985 |
| *A. cephalotes* | SES040120-01 | -8.720383333 | -35.84425 | BR: Frei Caneca | EU847865,EU847986 |
| *A. cephalotes* | SES040120-04 | -8.720383333 | -35.84425 | BR: Frei Caneca | EU847866,EU847987 |
| *A. cephalotes* | SES040121-03 | -8.720383333 | -35.84425 | BR: Frei Caneca | EU847867,EU847988 |
| *A. cephalotes* | SES040123-01 | -14.75536111 | -39.23255556 | BR: CEPLAC | EU847868,EU847989 |
| *A. cephalotes* | SES040123-03 | -14.75536111 | -39.23255556 | BR: CEPLAC | EU847869,EU847990 |
| *A. cephalotes* | SES040123-06 | -14.75536111 | -39.23255556 | BR: CEPLAC | EU847870,EU847991 |
| *A. cephalotes* | SES040123-07 | -14.75536111 | -39.23255556 | BR: CEPLAC | EU847871,EU847992 |
| *A. cephalotes* | SES040124-01 | -14.83975 | -39.02688889 | BR: Ubaitaba | EU847872,EU847993 |
| *A. cephalotes* | SES040124-02 | -14.83975 | -39.02688889 | BR: Ubaitaba | EU847873,EU847994 |
| *A. cephalotes* | SES040124-03 | -14.83975 | -39.02688889 | BR: Ubaitaba | EU847874,EU847995 |
| *A. cephalotes* | SES040125-01 | -14.41355556 | -39.33088889 | BR: Fazenda de Cascata | EU847875,EU847996 |
| *A. cephalotes* | SES040125-02 | -14.41355556 | -39.33088889 | BR: Fazenda de Cascata | EU847876,EU847997 |
| *A. cephalotes* | SES040129-03 | -9.75642 | -55.86272 | BR: Alta Floresta | EU847877,EU847998 |
| *A. cephalotes* | SES040129-06 | -9.75642 | -55.86272 | BR: Alta Floresta | EU847878,EU847999 |
| *A. cephalotes* | SES040131-04 | -10.05337 | -55.43224 | BR: Alta Floresta | EU847879,EU848000 |
| *A. cephalotes* | SES040131-06 | -10.05337 | -55.43224 | BR: Alta Floresta | EU847880,EU848001 |
| *A. cephalotes* | SES040131-12 | -9.86249 | -56.07603 | BR: Alta Floresta | EU847881,EU848002 |
| *A. cephalotes* | SES040131-13 | -9.86249 | -56.07603 | BR: Alta Floresta | EU847882,EU848003 |
| *A. cephalotes* | SES040204-03 | -1.68755 | -48.54977 | BR: Belem | EU847883,EU848004 |
| *A. cephalotes* | SES040208-06 | 0.62059 | -51.69178 | BR: Macapa | EU847884,EU848005 |
| *A. cephalotes* | SES040208-10 | 0.60082 | -51.75435 | BR: Macapa | EU847885,EU848006 |
| *A. cephalotes* | SES040214-03 | -1.90357 | -54.64113 | BR: Alenquer | EU847886,EU848007 |
| *A. cephalotes* | SES040215-03 | -1.92631 | -54.63676 | BR: Alenquer | EU847887,EU848008 |
| *A. cephalotes* | SES040220-01 | -3.65538 | -60.26173 | BR: Carreiro da Varzea | EU847888,EU848009 |
| *A. cephalotes* | SES040305-01 | 5.10819 | -60.82989 | VZ: Kukenan Camp | EU847889,EU848010 |
| *A. cephalotes* | SES040528-01 | -12.90274 | -71.42362 | PU: Pillcopata | EU847890,EU848011 |
| *A. cephalotes* | SES040528-06 | -12.90274 | -71.42362 | PU: Pillcopata | EU847891,EU848012 |
| *A. cephalotes* | SES040528-08 | -12.90274 | -71.42362 | PU: Pillcopata | EU847892,EU848013 |
| *A. cephalotes* | SES040530-03 | -12.82841 | -71.36393 | PU: Salvación | EU847893,EU848014 |
| *A. cephalotes* | SES040605-01 | -12.56895 | -70.1002 | PU: CICRA | EU847894,EU848015 |
| *A. cephalotes* | SES040605-03 | -12.56895 | -70.1002 | PU: CICRA | EU847895,EU848016 |
| *A. cephalotes* | SES040605-06 | -12.56895 | -70.1002 | PU: CICRA | EU847896,EU848017 |
| *A. cephalotes* | SES040605-07 | -12.56895 | -70.1002 | PU: CICRA | EU847897,EU848018 |
| *A. cephalotes* | SES040606-01 | -12.56895 | -70.1002 | PU: CICRA | EU847898,EU848019 |
| *A. cephalotes* | SES040607-01 | -12.56895 | -70.1002 | PU: CICRA | EU847899,EU848020 |
| *A. cephalotes* | SES040609-01 | -12.56895 | -70.1002 | PU: CICRA | EU847900,EU848021 |
| *A. cephalotes* | SES040609-05 | -12.56895 | -70.1002 | PU: CICRA | EU847901,EU848022 |
| *A. cephalotes* | SES040613-02 | -3.44336 | -72.84978 | PU: Explorama Lodge | EU847902,EU848023 |
| *A. cephalotes* | SES040615-03 | -3.44336 | -72.84978 | PU: Explorama Lodge | EU847903,EU848024 |
| *A. cephalotes* | SES040615-05 | -3.44336 | -72.84978 | PU: Explorama Lodge | EU847904,EU848025 |
| *A. cephalotes* | SES040616-02 | -3.2489 | -72.90908 | PU: ACTS | EU847905,EU848026 |
| *A. cephalotes* | SES040616-03 | -3.2489 | -72.90908 | PU: ACTS | EU847906,EU848027 |
| *A. cephalotes* | SES040617-02 | -3.2489 | -72.90908 | PU: ACTS | EU847907,EU848028 |
| *A. cephalotes* | SES040617-03 | -3.2489 | -72.90908 | PU: ACTS | EU847908,EU848029 |
| *A. cephalotes* | SES050730-04 | 10.34846 | -67.68436 | VZ: Rancho Grande Stn. | EU847909,EU848030 |
| *A. cephalotes* | SES050801-04 | 10.34846 | -67.68436 | VZ: PN Henri Pittier | EU847910,EU848031 |
| *A. cephalotes* | SES050801-05 | 10.34846 | -67.68436 | VZ: PN Henri Pittier | EU847911,EU848032 |
| *A. cephalotes* | SES050801-06 | 10.34846 | -67.68436 | VZ: PN Henri Pittier | EU847912,EU848033 |
| *A. cephalotes* | SES050803-01 | 9.25677 | -70.85779 | VZ: Monte Carmelo | EU847913,EU848034 |
| *A. cephalotes* | SES050803-02 | 9.25677 | -70.85779 | VZ: Monte Carmelo | EU847914,EU848035 |
| *A. cephalotes* | SES050803-03 | 9.00528 | -71.08352 | VZ: Monte Aventino | EU847915,EU848036 |
| *A. cephalotes* | SES050803-04 | 9.00528 | -71.08352 | VZ: Monte Aventino | EU847916,EU848037 |
| *A. cephalotes* | SES050803-05 | 9.00528 | -71.08352 | VZ: Monte Aventino | EU847917,EU848038 |
| *A. cephalotes* | SES050803-06 | 9.00528 | -71.08352 | VZ: Monte Aventino | EU847918,EU848039 |
| *A. cephalotes* | SES050804-02 | 8.73499 | -71.44686 | VZ: Parque La Palmita | EU847919,EU848040 |
| *A. cephalotes* | SES050804-03 | 8.73499 | -71.44686 | VZ: Parque La Palmita | EU847920,EU848041 |
| *A. cephalotes* | SES050804-04 | 8.73499 | -71.44686 | VZ: Parque La Palmita | EU847921,EU848042 |
| *A. cephalotes* | SES050807-11 | 8.84314 | -70.49066 | VZ: Calderas | EU847922,EU848043 |
| *A. cephalotes* | SES050817-02 | 8.13954 | -61.68963 | VZ: Camp Rio Grande | EU847923,EU848044 |
| *A. cephalotes* | SES050817-03 | 8.13954 | -61.68963 | VZ: Camp Rio Grande | EU847924,EU848045 |
| *A. cephalotes* | SSP0304XX-XX | 20.4 | -88.36 | MX: Chichén Itzá | EU847925,EU848046 |
| *A. cephalotes* | SV030618-09 | -0.63825 | -76.14931667 | EC: Tiputini | EU847926,EU848047 |
| *A. cephalotes* | Talamanca 4 | 9.640666667 | -82.6928 | CR: Pto Viejo Talamanca 4 |  |
| *A. cephalotes* | Talamanca 5 | 9.64005 | -82.69256667 | CR: Pto Viejo Talamanca 5 |  |
| *A. cephalotes* | Temascal | 18.23121667 | -96.4189 | MX: Temascal |  |
| *A. cephalotes* | TIKAL | 17.13 | -89.24 | GT: Tikal | EU847927,EU848048 |
| *A. cephalotes* | UGM050719-03 | 4.559766667 | -52.2068 | FG: Amazon Nature Lodge | EU847928,EU848049 |
| *A. cephalotes* | UGM050727-01 | 4.5518 | -52.21196667 | FG: Amazon Nature Lodge | EU847929,EU848050 |
| *A. cephalotes* | UGM050727-02 | 4.557833333 | -52.2039 | FG: Amazon Nature Lodge | EU847930,EU848051 |
| *A. cephalotes* | UGM050727-03 | 4.498266667 | -52.05238333 | FG: Kaw Boat Landing | EU847931,EU848052 |
| *A. cephalotes* | UGM050727-04 | 4.55705 | -52.17618333 | FG: Amazon Nature Lodge | EU847932,EU848053 |
| *A. cephalotes* | UGM950108-05 | 10.683611 | -61.283333 | TR: Simla | EU847933,EU848054 |
| *A. cephalotes* | UGM950109-04 | 10.683611 | -61.283333 | TR: Simla | EU847934,EU848055 |
| *A. cephalotes* | UGM950111-02 | 10.683611 | -61.283333 | TR: Simla | EU847935,EU848056 |
| *A. cephalotes* | UGM950111-03 | 10.683611 | -61.283333 | TR: Simla | EU847936,EU848057 |
| *A. cephalotes* | UGM950113-07 | 10.683611 | -61.283333 | TR: Simla | EU847937,EU848058 |
| *A. cephalotes* | UGM950114-10 | 10.683611 | -61.283333 | TR: Simla | EU847938,EU848059 |
| *A. cephalotes* | Valle Estrella | 9.743916667 | -82.93256667 | CR: Valle de la Estrella |  |
| *A. cephalotes* | Westfalia 1 | 9.935983333 | -83.00621667 | CR: Westfalia 1 |  |
| *A. cephalotes* | Westfalia 2 | 9.9356 | -83.00588333 | CR: Westfalia 2 |  |
| *A. columbica* | AGH020623-14 | 9.6838 | -83.0429 | CR: Hitoy Cerere | EU847939,EU848060 |
| *A. laevigata* | A21 | -21.26 | -45.03 | BR: Lavras |  |
| *A. laevigata* | A22 | -21.503333 | -46.209166 | BR: Jardim |  |
| *A. laevigata* | A23 | -24.11 | -52.62 | BR: Farol |  |
| *A. laevigata* | A24 | -3.085277 | -60.01 | BR: Manaus |  |
| *A. laevigata* | AOMB170904-01 | -22.7 | -47.291388 | BR: Santa Barbara D'Oeste |  |
| *A. laevigata* | CR060816-01 | -2.48382 | -54.95919 | BR: Alter do Chao | EU848063,EU848093 |
| *A. laevigata* | CR060817-02 | -2.48382 | -54.95919 | BR: Alter do Chao | EU848064,EU848094 |
| *A. laevigata* | CR060819-04 | -2.53024 | -54.94993 | BR: Alter do Chao | EU848065,EU848095 |
| *A. laevigata* | CR060820-09 | -2.93728 | -54.98359 | BR: Floresta Nac. Tapajos | EU848066,EU848096 |
| *A. laevigata* | CR060903-07 | -2.8907 | -59.96969 | BR: EMBRAPA | EU848067,EU848097 |
| *A. laevigata* | CR060904-25 | -2.89824 | -59.9903 | BR: EMBRAPA | EU848068,EU848098 |
| *A. laevigata* | E1142 | -22.9 | -48.46 | BR: Botucatu |  |
| *A. laevigata* | E121 | -21.266666 | -43.483333 | BR: Viçosa |  |
| *A. laevigata* | E633 | 19 | -48.31 | BR: Uberlandia |  |
| *A. laevigata* | E669 | -16.366666 | -48.466666 | BR: Brasilia |  |
| *A. laevigata* | E677 | -16.84 | -53.01 | BR: Araguainha |  |
| *A. laevigata* | E678 | -15.873611 | -49.606388 | BR: Serranopolis |  |
| *A. laevigata* | E959 | -2.9 | -44.45 | BR: São Luis |  |
| *A. laevigata* | SES040127-01 | -14.47869444 | -56.16755556 | BR: Posto Gil | EU848069,EU848099 |
| *A. laevigata* | SES040127-02 | -13.75377778 | -56.05347222 | BR: Mato Grosso 1 |  |
| *A. laevigata* | SES040127-05 | -12.08227778 | -55.51755556 | BR: Mato Grosso 3 |  |
| *A. laevigata* | SES040201-02 | -11.21058 | -55.30494 | BR: Itauba | EU848070,EU848100 |
| *A. laevigata* | SES040201-03 | -12.09205 | -55.51761 | BR: Mato Grosso 2 |  |
| *A. laevigata* | SES040207-01 | 1.43379 | -50.89864 | BR: Tartarugalzinho | EU848071,EU848101 |
| *A. laevigata* | SES040207-03 | 0.73981 | -51.33325 | BR: Ferreira Gomes | EU848072,EU848102 |
| *A. laevigata* | SES040207-04 | 0.73981 | -51.33325 | BR: Ferreira Gomes | EU848073,EU848103 |
| *A. laevigata* | SES040208-04 | 0.6772 | -51.50669 | BR: Amapa 3 |  |
| *A. laevigata* | SES040209-01 | 0.17151 | -51.51285 | BR: Amapa 2 |  |
| *A. laevigata* | SES040209-07 | 0.16766 | -51.12546 | BR: Amapa 1 | EU848074,EU848104 |
| *A. laevigata* | SES040212-07 | -2.82138 | -54.90031 | BR: Santerem |  |
| *A. laevigata* | SES040212-09 | -2.93924 | -54.9285 | BR: Santerem |  |
| *A. laevigata* | SES040218-02 | -2.69491 | -59.73894 | BR: Manaus | EU848075,EU848105 |
| *A. laevigata* | SES040218-04 | -2.69491 | -59.73894 | BR: Manaus | EU848076,EU848106 |
| *A. laevigata* | SES040218-07 | -2.61851 | -59.61978 | BR: Manaus |  |
| *A. laevigata* | SES040221-02 | -2.31131 | -60.02489 | BR: Manaus | EU848077,EU848107 |
| *A. laevigata* | SES040305-02 | 5.10819 | -60.82989 | VZ: Kukenan Camp |  |
| *A. laevigata* | SES050807-16 | 8.77066 | -70.42052 | VZ: Barinitas | EU848078,EU848108 |
| *A. laevigata* | SES050807-17 | 8.77066 | -70.42052 | VZ: Barinitas | EU848079,EU848109 |
| *A. laevigata* | SES050807-18 | 8.77066 | -70.42052 | VZ: Barinitas | EU848080,EU848110 |
| *A. laevigata* | SES050814-02 | 8.82739 | -64.14829 | VZ: El Tigre | EU848081,EU848111 |
| *A. laevigata* | SES050814-08 | 8.22513 | -63.50855 | VZ: Soledad | EU848082,EU848112 |
| *A. laevigata* | SES050814-10 | 8.22513 | -63.50855 | VZ: Soledad | EU848083,EU848113 |
| *A. laevigata* | SES050814-11 | 8.22513 | -63.50855 | VZ: Soledad | EU848084,EU848114 |
| *A. laevigata* | SES050814-13 | 8.22513 | -63.50855 | VZ: Soledad | EU848085,EU848115 |
| *A. laevigata* | SES050816-01 | 8.09461 | -63.20366 | VZ: Cd. Bolivar-Cd. Guayana 1 | EU848086,EU848116 |
| *A. laevigata* | SES050816-02 | 8.22086 | -62.85172 | VZ: Ciudad Guayana 1 |  |
| *A. laevigata* | SES050816-03 | 8.23616 | -62.83675 | VZ: Ciudad Guayana 2 |  |
| *A. laevigata* | SES050816-04 | 8.25965 | -62.81322 | VZ: Ciudad Guayana 3 | EU848087,EU848117 |
| *A. laevigata* | SES050816-05 | 8.25965 | -62.81322 | VZ: Ciudad Guayana 3 | EU848088,EU848118 |
| *A. laevigata* | SES050816-06 | 8.25965 | -62.81322 | VZ: Ciudad Guayana 3 | EU848089,EU848119 |
| *A. laevigata* | SES050818-01 | 7.88762 | -63.59712 | VZ: Ciudad Bolívar 1 |  |
| *A. laevigata* | SES050818-05 | 8.21699 | -62.85557 | VZ: Cd. Bolivar-Cd. Guayana 2 |  |
| *A. laevigata* | SES050818-06 | 8.73389 | -62.38002 | VZ: Temblador | EU848090,EU848120 |
| *A. laevigata* | SES050818-08 | 8.73389 | -62.38002 | VZ: Temblador | EU848091,EU848121 |
| *A. laevigata* | SES050819-01 | 9.00554 | -62.66763 | VZ: Temblador |  |
| *A. laevigata* | SES050819-02 | 8.97284 | -62.75017 | VZ: Temblador | EU848092,EU848122 |
| *A. laevigata* | SES050822-01 | 6.2465 | -62.85368 | VZ: Canaima |  |
| *A. mexicana* | GUADALUPE | ? | ? | MX: Guadalupe | EU847940,EU848061 |
| *A. sexdens* | Alenquer 3 | -1.90357 | -54.64113 | BR: Alenquer 3 |  |
| *A. sexdens* | CR060807-01 | -1.453502778 | -48.47670833 | BR: Belem 2 | EU848123,EU848169 |
| *A. sexdens* | CR060817-05 | -2.48382 | -54.95919 | BR: Alter do Chao | EU848124,EU848170 |
| *A. sexdens* | SES020520-02 | 9.369722 | -79.931944 | PA: Gamboa |  |
| *A. sexdens* | SES040120-02 | -8.720383333 | -35.84425 | BR: Frei Caneca |  |
| *A. sexdens* | SES040124-07 | -14.18722222 | -39.65983333 | BR: Bahia 2 | EU848125,EU848171 |
| *A. sexdens* | SES040124-09 | -14.09619444 | -39.78102778 | BR: Bahia 3 | EU848126,EU848172 |
| *A. sexdens* | SES040125-05 | -14.20027778 | -39.81586111 | BR: Bahia 1 | EU848127,EU848173 |
| *A. sexdens* | SES040125-06 | -14.20027778 | -39.81586111 | BR: Bahia 1 | EU848128,EU848174 |
| *A. sexdens* | SES040127-04 | -12.08227778 | -55.51755556 | BR: Sinop |  |
| *A. sexdens* | SES040128-01 | -9.89753 | -56.09404 | BR: Alta Floresta 3 |  |
| *A. sexdens* | SES040129-01 | -9.895055556 | -55.90791667 | BR: Alta Floresta 4 |  |
| *A. sexdens* | SES040130-04 | -9.5746 | -56.01333 | BR: Alta Floresta 6 |  |
| *A. sexdens* | SES040131-01 | -10.05337 | -55.43224 | BR: Alta Floresta 2 |  |
| *A. sexdens* | SES040131-11 | -10.06242 | -55.59001 | BR: Alta Floresta 1 | EU848129,EU848175 |
| *A. sexdens* | SES040131-14 | -9.86249 | -56.07603 | BR: Alta Floresta 5 |  |
| *A. sexdens* | SES040201-08 | -13.74634 | -56.05287 | BR: Novo Motum |  |
| *A. sexdens* | SES040204-04 | -1.416666 | -48.417555 | BR: Belem 3 |  |
| *A. sexdens* | SES040205-05 | -1.68755 | -48.54977 | BR: Belem 1 |  |
| *A. sexdens* | SES040208-02 | 0.6772 | -51.50669 | BR: Amapa 2 | EU848130,EU848176 |
| *A. sexdens* | SES040208-05 | 0.67436 | -51.53008 | BR: Amapa 1 |  |
| *A. sexdens* | SES040212-06 | -2.64342 | -54.78015 | BR: Santerem 2 | EU848131,EU848177 |
| *A. sexdens* | SES040213-03 | -2.55767 | -54.72733 | BR: Santerem 4 | EU848132,EU848178 |
| *A. sexdens* | SES040214-10 | -1.91676 | -54.62662 | BR: Alenquer 2 |  |
| *A. sexdens* | SES040215-07 | -1.92631 | -54.63676 | BR: Alenquer 1 | EU848133,EU848179 |
| *A. sexdens* | SES040215-08 | -1.92631 | -54.63676 | BR: Alenquer 1 | EU848134,EU848180 |
| *A. sexdens* | SES040215-10 | -1.92631 | -54.63676 | BR: Alenquer 1 | EU848135,EU848181 |
| *A. sexdens* | SES040216-03 | -2.58981 | -54.57096 | BR: Santerem 3 | EU848136,EU848182 |
| *A. sexdens* | SES040216-05 | -2.66756 | -54.6574 | BR: Santerem 1 |  |
| *A. sexdens* | SES040221-01 | -2.31131 | -60.02489 | BR: Manaus | EU848137,EU848183 |
| *A. sexdens* | SES040606-03 | -12.56895 | -70.1002 | PU: CICRA | EU848138,EU848184 |
| *A. sexdens* | SES040613-01 | -3.44336 | -72.84978 | PU: Explorama Lodge | EU848139,EU848185 |
| *A. sexdens* | SES040614-01 | -3.44336 | -72.84978 | PU: Explorama Lodge | EU848140,EU848186 |
| *A. sexdens* | SES040614-02 | -3.44336 | -72.84978 | PU: Explorama Lodge | EU848141,EU848187 |
| *A. sexdens* | SES040614-03 | -3.44336 | -72.84978 | PU: Explorama Lodge | EU848142,EU848188 |
| *A. sexdens* | SES040615-01 | -3.44336 | -72.84978 | PU: Explorama Lodge | EU848143,EU848189 |
| *A. sexdens* | SES040618-01 | -3.2489 | -72.90908 | PU: ACTS | EU848144,EU848190 |
| *A. sexdens* | SES040619-03 | -3.2489 | -72.90908 | PU: ACTS | EU848145,EU848191 |
| *A. sexdens* | SES050728-02 | 10.27306 | 67.61294 | VZ: Maracay | EU848146,EU848192 |
| *A. sexdens* | SES050728-08 | 10.27306 | 67.61294 | VZ: Maracay | EU848147,EU848193 |
| *A. sexdens* | SES050802-01 | 9.28387 | -70.86011 | VZ: Monte Carmelo | EU848148,EU848194 |
| *A. sexdens* | SES050802-02 | 9.28387 | -70.86011 | VZ: Monte Carmelo | EU848149,EU848195 |
| *A. sexdens* | SES050814-01 | 8.85397 | -64.204 | VZ: El Tigre 2 | EU848150,EU848196 |
| *A. sexdens* | SES050814-06 | 8.82739 | -64.14829 | VZ: El Tigre 1 | EU848151,EU848197 |
| *A. sexdens* | SES050815-01 | 8.14594 | -63.55125 | VZ: Ciudad Bolivar 2 | EU848152,EU848198 |
| *A. sexdens* | SES050815-02 | 8.13762 | -63.54602 | VZ: Ciudad Bolivar 1 |  |
| *A. sexdens* | SES050818-04 | 8.21699 | -62.85557 | VZ: Cd. Bolivar-Cd. Guayana |  |
| *A. sexdens* | SES050824-01 | 6.2465 | -62.85368 | VZ: Canaima | EU848153,EU848199 |
| *A. sexdens* | UGM050721-02 | 4.92245 | -52.28185 | FG: Montjoly 3 | EU848154,EU848200 |
| *A. sexdens* | UGM050721-05 | 4.886016667 | -52.26111667 | FG: Mont Rorora | EU848155,EU848201 |
| *A. sexdens* | UGM050723-01 | 4.944683333 | -52.31465 | FG: Montjoly 5 |  |
| *A. sexdens* | UGM050723-05 | 4.935983333 | -52.28626667 | FG: Montjoly 4 |  |
| *A. sexdens* | UGM050723-07 | 4.912566667 | -52.27195 | FG: Montjoly 1 |  |
| *A. sexdens* | UGM050723-08 | 4.885116667 | -52.26281667 | FG: Montjoly 2 | EU848156,EU848202 |
| *A. sexdens* | UGM050723-09 | 4.885116667 | -52.26281667 | FG: Montjoly | EU848157,EU848203 |
| *A. sexdens* | UGM050724-01 | 4.823033333 | -52.3643 | FG: Cayenne Airport | EU848158,EU848204 |
| *A. sexdens* | UGM050724-09 | 5.0709 | -52.5439 | FG: Tonate-Kourou Road |  |
| *A. sexdens* | UGM050724-10 | 5.159433333 | -52.67355 | FG: Mt. Pariacabo | EU848159,EU848205 |
| *A. sexdens* | UGM050725-01 | 5.239266667 | -52.90688333 | FG: Kourou-St.Laurent 1 | EU848160,EU848206 |
| *A. sexdens* | UGM050725-02 | 5.239266667 | -52.90688333 | FG: Kourou-St.Laurent 1 | EU848161,EU848207 |
| *A. sexdens* | UGM050725-03 | 5.239266667 | -52.90688333 | FG: Kourou-St.Laurent 1 | EU848162,EU848208 |
| *A. sexdens* | UGM050725-05 | 5.266383333 | -52.91666667 | FG: Kourou-St.Laurent 2 |  |
| *A. sexdens* | UGM050725-05 | 5.266383333 | -52.91666667 | FG: Kourou-St.Laurent 2 | EU848163,EU848209 |
| *A. sexdens* | UGM050725-09 | 5.480766667 | -53.5649 | FG: Kourou-St.Laurent 4 | EU848164,EU848210 |
| *A. sexdens* | UGM050726-10 | 5.48925 | -54.00153333 | FG: St. Laurent 2 | EU848165,EU848211 |
| *A. sexdens* | UGM050726-11 | 5.48925 | -54.00153333 | FG: St. Laurent 2 | EU848166,EU848212 |
| *A. sexdens* | UGM050726-13 | 5.488083333 | -53.98501667 | FG: St. Laurent 1 | EU848167,EU848213 |
| *A. sexdens* | UGM050726-15 | 5.470383333 | -53.57206667 | FG: Kourou-St.Laurent 3 | EU848168,EU848214 |
| *A. texana* | BFL-2 | 30.2828 | -97.7804 | US: Brackenridge Field Lab | EU847941,EU848062 |

Table S7: List of all samples used, their geographic locations, and GenBank Accession numbers for samples used in molecular analyses (BR=Brazil; BZ=Belize; CO=Colombia; CR=Costa Rica; EC=Ecuador; FG=French Guiana; GT=Guatemala; GU=Guyana; MX=Mexico; PA=Panama; PU=Peru; TR=Trinidad; US=United States; VZ=Venezuela).
