# Supplementary material for: Paleodistributions and Comparative Molecular Phylogeography of Leafcutter Ants (Atta spp.) Provide New Insight into the Origins of Amazonian Diversity
Source: PLoS One. 2008 Jul 23;3(7):e2738. doi: 10.1371/journal.pone.0002738 (PMC2447876; doi:10.1371/journal.pone.0002738)
Supplement: References S1 — (0.02 MB DOC) [file pone.0002738.s008.doc]

S1. Moreau, C.S., Bell, C.D., Vila, R., Archibald, S.B., Pierce, N.E. (2006) Phylogeny of the ants: Diversification in the age of angiosperms. Science 312: 101-104.
